# Single-cell and spatial transcriptomic analysis reveals tumor cell heterogeneity and underlying molecular program in colorectal cancer

**DOI:** 10.3389/fimmu.2025.1556386

**Published:** 2025-03-12

**Authors:** Teng Wang, Zhaoming Chen, Wang Wang, Heng Wang, Shenglong Li

**Affiliations:** ^1^ Department of Bioinformatics, School of Basic Medical Sciences, Chongqing Medical University, Chongqing, China; ^2^ Department of Immunology, School of Basic Medical Sciences, Chongqing Medical University, Chongqing, China; ^3^ Chongqing Key Laboratory of Tumor Immune Regulation and Immune Intervention, Chongqing Medical University, Chongqing, China

**Keywords:** colorectal cancer, tumor heterogeneity, prognosis, therapy, single-cell transcriptomics, spatial transcriptomics

## Abstract

**Background:**

Colorectal cancer (CRC) is a highly heterogeneous tumor, with significant variation in malignant cells, posing challenges for treatment and prognosis. However, this heterogeneity offers opportunities for personalized therapy.

**Methods:**

The consensus non-negative matrix factorization algorithm was employed to analyze single-cell transcriptomic data from CRC, which helped identify malignant cell expression programs (MCEPs). Subsequently, a crosstalk network linking MCEPs with immune/stromal cell trajectory development was constructed using Monocle3 and NicheNet. Additionally, bulk RNA-seq data were utilized to systematically explore the relationships between MCEPs, clinical features, and genetic mutations. A prognostic model was then established through Lasso and Cox regression analyses, integrating clinical data into a nomogram for personalized risk prediction. Furthermore, key genes associated with MCEPs and their potential therapeutic targets were identified using protein-protein interaction networks, followed by molecular docking to predict drug-binding affinity.

**Results:**

We classified CRC malignant cell transcriptional states into eight distinct MCEPs and successfully constructed crosstalk networks between these MCEPs and immune or stromal cells. A prognostic model containing 15 genes was developed, demonstrating an AUC greater than 0.8 for prognostic evaluation over 1 to 10 years when combined with clinical features. A key drug target gene TIMP1 was identified, and several potential targeted drugs were discovered.

**Conclusion:**

This study demonstrated that characterization of the malignant cell transcriptional programs could effectively reveal the biological features of highly heterogeneous tumors like CRC and exhibit significant potential in tumor prognosis assessment. Our research provides new theoretical and practical directions for CRC prognosis and targeted therapy.

## 1 Introduction

Colorectal cancer (CRC) is one of the three most common cancers worldwide and the second leading cause of cancer-related deaths, driven by its profound molecular and cellular heterogeneity (1–3). CRC is primarily classified into two genetic subtypes—chromosomal instability (CIN) and microsatellite instability (MSI)—with distinct biological behaviors and therapeutic responses (4–7). Immune checkpoint blockade (ICB) therapy has shown efficacy in advanced MSI-H tumors, yet most patients remain unresponsive, underscoring the need for novel biomarkers (8–10). Molecular subtyping approaches, such as the Consensus Molecular Subtypes (CMS) classification, integrate bulk transcriptomic and genomic data to stratify CRC into four prognostic subtypes (CMS1-4) (11). However, these bulk-level analyses fail to resolve the continuum of malignant cell states or their dynamic crosstalk with the tumor microenvironment (TME) (12, 13).

Recent advances in single-cell and spatial transcriptomics have revolutionized cancer research by enabling high-resolution dissection of tumor heterogeneity. Single-cell RNA sequencing (scRNA-seq) and ATAC-seq reveal transcriptional and epigenetic diversity within malignant cells, while spatial technologies map cellular interactions in TME niches (14–17). Despite these advances, existing studies often categorize malignant cells into discrete subtypes or focus on isolated TME components, neglecting the continuum of transcriptional plasticity and bidirectional stromal-immune interactions (18–20). Traditional methods like PCA or clustering impose rigid structures on transcriptional data: PCA reduces variance to orthogonal components but obscures transitional states, while clustering forces discrete boundaries on inherently continuous programs. In contrast, consensus non-negative matrix factorization (cNMF) decodes continuous transcriptional dynamics, as demonstrated by its ability to resolve plastic cell states in lung cancer (21).

To advance beyond these limitations, this study integrates single-cell and spatial multi-omics data, applying cNMF to decode CRC heterogeneity. We identified eight continuous transcriptional programs (MCEPs) in malignant cells, encompassing dynamic phenotypes such as hypoxia adaptation, partial EMT plasticity, and glandular differentiation. By combining spatial co-localization with pseudotime trajectory analysis of stromal and immune cells, we uncovered how MCEPs remodel the TME through specific regulatory nodes (e.g., TGFB1-mediated fibroblast activation, HMGB2-dependent angiogenesis). Furthermore, we developed a prognostic model integrating MCEP-TME interactions, validated through protein-protein network analysis and experimental databases to prioritize therapeutic targets.

The eight MCEPs delineate critical biological dimensions in colorectal cancer progression (1): Inflammatory-Hypoxia Stress Program (IHS-P) coordinates hypoxic adaptation and immune modulation within immune-enriched niches (2); Wnt Signaling Stress Program (Wnt-S-P) drives canonical Wnt activation in tumor cores (3); Proliferation Stress Program (PS-P) governs cell cycle progression through MYC/mTORC1 signaling (4); Inflammatory Epithelial pEMT Program (IE-pEMT-P) bridges interferon responses with partial EMT plasticity (5); Intermediate pEMT Program (I-pEMT-P) mediates TGFB1-dependent stromal activation (6); Mesenchymal pEMT Program (M-pEMT-P) executes ECM remodeling in stromal compartments (7); Cell Cycle Program (CC-P) regulates pan-tumoral mitotic processes (8); Glandular Secretion Program (GS-P) maintains epithelial differentiation near normal tissues. This framework deciphers CRC heterogeneity through malignant cell state dynamics and their spatial-ecological networks, enabling prognostic prediction and therapeutic target discovery for precision oncology.

## 2 Materials and methods

### 2.1 Download and preprocessing of single-cell and spatial transcriptomics sequencing data

Single-cell RNA sequencing data were processed using Seurat (v5.1.0) with rigorous quality control. Three publicly available human colorectal cancer datasets were analyzed: GSE166555 (13 tumors, 12 normals) (22), GSE200997 (16 tumors, 7 normals) (23) from the Gene Expression Omnibus (GEO) database (https://www.ncbi.nlm.nih.gov/geo/), and syn26844071 (141 tumors, 39 normals) (24) from the Synapse database (https://www.synapse.org/). Doublets were removed using Scrublet (v0.2.3), followed by gene/cell filtering criteria: genes detected in ≥3 cells, cells expressing ≥250 genes, UMI counts <15,000, mitochondrial gene percentage <20%, and erythrocyte gene ratio <1%.

Spatial transcriptomics data were obtained from the 10x Genomics Visium HD platform (8 μm resolution) and downloaded from the official 10x Genomics website (https://www.10xgenomics.com/), comprising a total of three samples (25). Quality control was performed on the spatial transcriptomics data, with spots retained for downstream analysis meeting the following thresholds: detection of ≥10 genes, UMI counts >20, and mitochondrial gene ratio <25%.

### 2.2 Cell annotation for single-cell and spatial transcriptomics data

scRNA-seq data underwent log-normalization and identification of highly variable genes (vst method). Batch correction was performed using Harmony (v0.1.0). Cell types were annotated through a two-step approach: 1) Initial classification using SingleR (v2.6.0) and CellTypist (v1.6.3) with canonical markers; 2) Refinement via secondary dimensionality reduction and iterative CellTypist-based annotation, followed by removal of misclassified cells.

For spatial data, we implemented memory-efficient processing by subsampling 50,000 points using SketchData. Cell type deconvolution was performed using RCTD (v2.2.1) with scRNA-seq data as reference. Each spatial sample underwent independent dimensionality reduction and annotation.

### 2.3 Identification of malignant epithelial cells and gene expression program profiling

Epithelial cells were isolated from the full cell atlas and subjected to chromosomal copy number variation (CNV) analysis using inferCNV (v1.18.1), with normal colorectal epithelial cells as the reference. A CNV score matrix was generated, and unsupervised K-means clustering partitioned cells into malignant or normal clusters based on CNV-driven cluster purity.

For malignant cell subtyping, consensus high-variance genes were identified through 200 iterations of 75% subsampling. Genes recurrently ranked among the top 2,500 highly variable genes in ≥150 iterations were retained. These genes underwent non-negative matrix factorization (cNMF) to decompose the expression matrix into gene expression programs (GEPs) and their corresponding activity scores. The optimal number of GEPs was determined by minimizing reconstruction error and maximizing stability via elbow plot analysis.

To define high-weight genes within each MCEP, genes were ranked by their absolute weights in the cNMF gene coefficient matrix. The top 100 genes per program, exhibiting the strongest association with each transcriptional module, were selected for downstream spatial mapping. Spatial enrichment scores for these gene sets were computed using the AUCell R package (v1.24.0), enabling visualization of MCEP distribution patterns across tissue sections.

### 2.4 Pseudotime analysis

Developmental trajectories were reconstructed using Monocle3 (v1.3.5) with UMAP for dimensionality reduction. Cell subtypes were pre-annotated through immune and stromal cell clustering, which revealed preliminary developmental hierarchies. To resolve ambiguous differentiation origins arising from complex branching trajectories, we implemented a hybrid strategy for root node selection (1): For lineages with biologically established progenitor-differentiated cell relationships (e.g., T cell and B cell hierarchies), root nodes were manually assigned to progenitor states based on canonical marker expression and prior biological knowledge (2); For cell types lacking definitive developmental origins, root nodes were computationally determined by selecting the subpopulation with the highest transcriptional immaturity index, as quantified by CytoTRACE2 (v1.0.0). Trajectory-associated genes were identified using Monocle3’s graph_test function with “neighbor_graph=principal_graph” to evaluate gene expression dynamics along reconstructed paths.

### 2.5 Expression program crosstalk networks

Intercellular crosstalk networks were constructed by defining trajectory-associated genes (Moran’s |I| > 0.25, q < 0.05) from each malignant cell population as target gene sets. For each MCEP, the top 100 weighted genes in expression programs were selected as candidate regulators. Ligand-target interactions were predicted using NicheNet (v2.1.5), generating regulatory potential matrices where malignant cell regulators were prioritized based on their capacity to modulate target gene sets. Potential interactions in the lowest tertile of regulatory scores were nullified to eliminate spurious associations. Final immune and stromal interaction networks were reconstructed in Cytoscape (v3.10.2) using thresholded matrices for edge weighting.

### 2.6 Bulk sequencing data sources

Bulk RNA-seq data and simple nucleotide variation (SNV) data for colorectal cancer were obtained from The Cancer Genome Atlas (TCGA) database (https://www.cancer.gov/ccg/research/genome-sequencing/tcga). Using the R package TCGAbiolinks (v2.30.4), we retrieved RNA-seq data from 581 colorectal cancer patients and 51 normal colorectal control samples, along with SNV data for 538 patients. Clinical data for TCGA patients and pan-cancer gene expression profiles were additionally acquired from the UCSC Xena database (https://xena.ucsc.edu/).

To complement TCGA data, gene expression microarray datasets and corresponding clinical information were downloaded from the GEO database. Datasets included GSE39582 (26), GSE17536 (27), GSE17537 (27), GSE29621 (28), GSE38832 (29), GSE143985 (30), and GSE161158 (31), all generated on the GPL570 platform. From GSE39582, GSE17536, GSE17537, GSE29621, and GSE38832, overall survival (OS) data were extracted. After filtering samples with missing survival time, status, or non-positive survival time, 573, 177, 55, 65, and 122 samples were retained, respectively. Disease-free survival (DFS) and recurrence/survival status data were obtained from GSE143985 and GSE161158. Following similar quality control, 91 and 174 samples were retained, respectively.

### 2.7 Differential and enrichment analyses

To further investigate the changes in expression program-related genes at the bulk level, we integrated two distinct gene cohorts: 1) the top 100 weighted genes from each MCEP module, and 2) computationally predicted target genes in the MCEP-immune/stromal cell crosstalk network. Differential gene expression analysis was performed on this merged gene set using bulk RNA-seq data from the TCGA cohort through the R package DESeq2 (version 1.42.1). Statistical significance was defined as absolute Fold Change > 1.5 and padj < 0.05. Gene Ontology (GO) and Kyoto Encyclopedia of Genes and Genomes (KEGG) pathway enrichment analyses were subsequently conducted on the identified differentially expressed genes (DEGs) using the clusterProfiler package (version 4.2.2) to characterize their functional roles.

### 2.8 Consensus clustering and intra-cluster comparison

Differentially expressed genes from TCGA were subjected to univariate Cox regression analysis (survival package v3.5-8, p<0.05) to identify survival-associated genes. Consensus clustering via ConsensusClusterPlus (v1.66.0) with 500 bootstraps (80% sample resampling) and K-means (Euclidean distance) identified optimal clusters (k=2-10) by evaluating consensus matrices and cumulative distribution functions (CDF). Subtype-specific survival differences were assessed by Kaplan-Meier analysis, while chi-square tests evaluated clinical characteristics (gender, age, stage). Mutation landscapes were visualized using maftools (v2.18.0), highlighting the top 15 recurrently mutated genes per subtype.

### 2.9 Construction of the prognostic model

Gene expression data were obtained from TCGA and seven GEO datasets (GSE39582, GSE17536, GSE17537, GSE29621, GSE38832, GSE143985, GSE161158). Batch effects were mitigated through z-score normalization followed by batch correction using the `removeBatchEffect` function (limma package v3.58.1). The TCGA and GSE39582 cohorts were partitioned into a training set (70% of samples) and an internal validation set (30%), while remaining datasets served as external validation cohorts.

To address feature redundancy, genes identified by univariate Cox regression (p < 0.05) were subjected to Lasso regression (glmnet v4.1-4) for dimensionality reduction. A stepwise backward Cox regression was then applied to optimize model complexity by minimizing the Akaike Information Criterion (AIC).

Risk scores were computed for all samples across training and validation cohorts. Survival differences between high- and low-risk groups (stratified by median risk scores) were evaluated using Kaplan-Meier analysis with log-rank tests. Predictive performance was quantified via time-dependent ROC curves and AUC values. Model robustness and clinical applicability were systematically validated across internal and external datasets using survival outcomes and AUC consistency.

### 2.10 Bulk immune landscape and calculation of single-cell and spatial risk scores

To explore the biological relevance of our prognostic model, we performed tumor immune microenvironment analysis on the TCGA cohort using the IOBR package (v0.99.9). Immune cell composition was quantified by integrating eight computational algorithms (MCPcounter, EPIC, xCell, CIBERSORT, IPS, quanTIseq, ESTIMATE, and TIMER). Spearman correlation analysis was then applied to evaluate associations among immune infiltration scores, prognostic feature gene expression, and sample risk scores.

For single-cell and spatial transcriptomic data, we adapted our risk scoring approach to address inherent data sparsity. Based on the regression coefficients from the linear prognostic model, feature genes were partitioned into two subsets: a positive-coefficient subset (PosRisk genes) and a negative-coefficient subset (NegRisk genes). The AddModuleScore function was employed to calculate PosRiskScore and NegRiskScore for each subset independently. Final RiskScore was derived as PosRiskScore minus NegRiskScore. This strategy enabled robust quantification of model-associated biological processes at cellular and spatial resolutions while mitigating technical limitations of sparse transcriptomic data.

### 2.11 Construction of a nomogram

Univariate Cox regression analysis was performed on TCGA cohort data to preliminarily identify variables (risk score, age, gender, tumor stage, and other clinical features) associated with overall survival. Subsequently, multivariate Cox regression analysis incorporating all candidate variables without prior feature selection was conducted to evaluate their independent prognostic contributions while adjusting for potential confounders.

A nomogram integrating the risk score and significant clinical predictors was developed using the regplot package (v1.1) to visualize survival probability estimates. Time-dependent receiver operating characteristic (ROC) analyses spanning 1-10 years were implemented to quantify predictive accuracy through area under the curve (AUC) calculations. Model calibration was validated using the rms package (v6.8-1) by comparing predicted versus observed survival probabilities via bootstrapped calibration curves (1,000 resamples). Clinical utility was further assessed through decision curve analysis (DCA) using the rmda package (v1.6), which quantified net benefits across threshold probabilities ranging from 0% to 100%. This comprehensive validation framework ensures methodological rigor and supports clinical translation of the prognostic model.

### 2.12 Key genes identification with malignant cell expression programs and drug screening

Differential expression analysis was performed on prioritized genes derived from malignant cell expression programs and their microenvironment-associated targets. Resultant genes were analyzed through the STRING database (https://cn.string-db.org/) to construct protein-protein interaction (PPI) networks, which were further visualized and analyzed in Cytoscape (v3.9.1). Core hub genes were systematically identified using the cytoHubba plugin (v0.1) with four topology-based algorithms: MNC, MCC, DMNC, and Degree.

Expression differences of candidate genes between tumor and adjacent normal tissues were statistically validated using the Wilcoxon rank-sum test. Immunohistochemical images from The Human Protein Atlas (HPA, https://www.proteinatlas.org/) were utilized as supporting evidence.

For therapeutic exploration, three-dimensional structures of key targets were retrieved from UniProt (https://www.uniprot.org/), and 2,391 FDA-approved small-molecule drugs were sourced from DrugBank (https://go.drugbank.com/). Structural data standardization was implemented using rdkit (v2023.9.6) and meeko (v0.5.1), followed by protein active site prediction via the Prankweb database (https://prankweb.cz/). Molecular docking simulations were executed with AutoDock Vina (v1.2.5), prioritizing compounds based on binding affinity (ΔG, kcal/mol). The top two ligands exhibiting optimal docking scores were selected for binding conformation visualization using PyMOL (v3.1.0a0).

### 2.13 Software and data analysis tools

Single-cell and spatial transcriptomic analyses were performed using R (v4.3.2), with the cNMF algorithm (https://github.com/dylkot/cNMF) implemented in Python (v3.8.19). Drug virtual screening was conducted using Python (v3.10.14). Data visualization was facilitated by R packages, including SCP (v0.5.6), ggplot2 (v3.5.1), and ComplexHeatmap (v2.18.0). Univariate and multivariate Cox regression analyses were executed using the survival package (v3.5-8), while time-dependent AUC values were computed with the timeROC package (v0.4). Kaplan-Meier survival curves were generated using the survminer package (v0.4.9).

## 3 Results

### 3.1 Identification of malignant cells and characterization of heterogeneous expression programs

**Figure 1:**
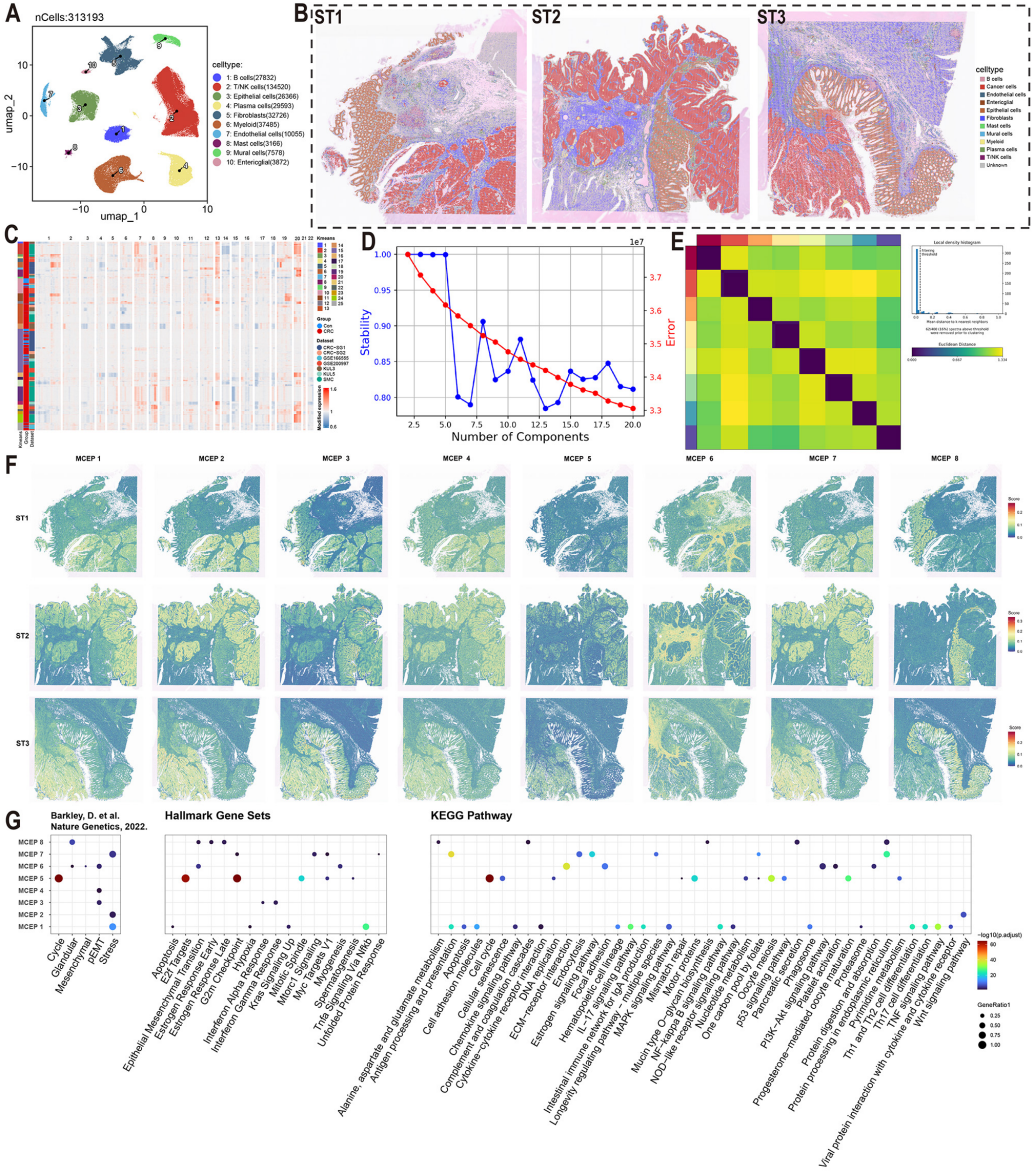
Functional characterization of malignant cell expression programs in colorectal cancer. **(A)** UMAP visualization of major cell types color-coded by cell lineage following quality control. **(B)** Spatial mapping of cell type distributions across three independent colorectal cancer specimens (ST1-3) using spatial transcriptomics. **(C)** Copy number variation (CNV) heatmap of epithelial cells stratified by k-means clustering (left panel). Tumor-derived cells (red) and normal counterparts (blue) are segregated based on chromosomal amplification (red) and deletion (blue) patterns. **(D)** Model selection curve demonstrating the optimal number of expression programs determined by consensus non-negative matrix factorization (CNMF), balancing stability and reconstruction error. **(E)** Consensus matrix establishing robust program identification. **(F)** Spatial activation patterns of MCEPs across tumor sections (ST1-3). **(G)** Functional enrichment analysis integrating pan-cancer malignant cell states (Barkley et al.), Hallmark gene sets, and KEGG pathways.

In this study, we integrated single-cell transcriptomic data from three datasets (GSE166555, GSE200997, and syn26844071), comprising 58 normal colorectal samples and 170 CRC samples. Following rigorous quality control and dimensionality reduction, a total of 320,475 cells were classified into 10 major cell types: B cells, T/NK cells, epithelial cells, plasma cells, fibroblasts, myeloid cells, endothelial cells, mast cells, mural cells, and enteric glial cells. Among these, T/NK cells were the most abundant (135,789 cells), followed by myeloid cells and fibroblasts (**Figure 1A**, **Supplementary Figure S1**-**Supplementary Figure S2**, and **Supplementary Figure S3A-G**). These refined annotations were applied to three high-resolution spatial transcriptomic datasets (ST1, ST2, ST3), enabling the visualization of the spatial distribution of different cell types within colorectal cancer tumors (**Figure 1B**).

To further investigate CRC heterogeneity, epithelial cell data were extracted from the comprehensive cell atlas. To ensure the purity of the epithelial cells, we re-annotated them using the SingleR and CellTypist algorithms, removing incorrectly classified cells (**Supplementary Figure S4A-B**). CNV scoring was performed on epithelial cells from tumor samples using the inferCNV algorithm, with normal epithelial cells serving as the reference. K-means clustering of the CNV score matrix revealed that epithelial cells from normal samples predominantly clustered in clusters 10, 15, and 25, exhibiting no significant CNV alterations. In contrast, epithelial cells from tumor samples showed clear gene copy number alterations, distinguishing them as malignant cells (**Figure 1C**, **Supplementary Figure S4C**). Malignant epithelial cells were identified by excluding clusters 10, 15, and 25 from the tumor samples.

Given the high heterogeneity of CRC cells, traditional clustering methods were insufficient to fully capture their complexity. Therefore, we applied the cNMF algorithm, which demonstrated high stability and low error when set to eight expression programs (**Figure 1D**). Consensus analysis confirmed the robustness of these eight expression programs, with substantial consistency across repeated experiments and outliers identified using a threshold of 0.05 (**Figure 1E**, **Supplementary Figure S4D**). These eight stable expression programs effectively captured the transcriptional characteristics of malignant CRC cells, providing a reliable framework for further analysis of CRC heterogeneity.

To visualize the spatial distribution of these MCEPs, we applied the AUCell algorithm to spatial transcriptomic data, scoring each sample based on the top 100 weight genes of each program. Enrichment analysis of the top 100 weight genes from each program was conducted, primarily referencing a gene set from the study by Barkley, D. et al. on pan-cancer tumor cell heterogeneity, supplemented with enrichment results from Hallmark Gene Sets and KEGG Pathways (32). This analysis revealed that MCEP 1, 2, and 7 were associated with stress responses. MCEP 1 was enriched in pathways related to hypoxia, antigen processing and presentation, chemokine signaling, and IL-17 signaling, while MCEP 2 was enriched in Wnt signaling. MCEP 7 was enriched in cell proliferation-related pathways, including the G2M checkpoint, mTORC1 signaling, and Myc targets V1. These programs were categorized as Inflammatory-Hypoxia Stress Expression Program (IHS-P), Wnt Signaling Stress Expression Program (Wnt-S-P), and Proliferation Stress Expression Program (PS-P), respectively. The spatial distribution of these MCEPs showed that IHS-P was prevalent in malignant and immune cell-rich regions, while Wnt-S-P and PS-P were more confined to malignant cells (**Figures 1F, G**).

Additionally, MCEP 3, 4, and 6 were associated with pEMT states. MCEP 3 was enriched in pEMT states and interferon responses, with higher spatial scores observed in both malignant and normal epithelial cells. MCEP 6, enriched in mesenchymal, myogenesis, and ECM-receptor interaction pathways, displayed preferential spatial scores in the stromal compartment. Based on these findings, MCEPs 3, 4, and 6 were categorized as Inflammatory Epithelial-type pEMT Program (IE-pEMT-P), Intermediate Type pEMT Expression Program (I-pEMT-P), and Mesenchymal Type pEMT Expression Program (M-pEMT-P), respectively. The spatial distributions and enrichment results for these programs are shown in **Figures 1F, G**.

MCEP 5, enriched in cell cycle-related pathways such as Cell Cycle, E2F Targets, and G2M checkpoint, exhibited a dispersed spatial distribution across malignant and epithelial cells, and was categorized as the Cell Cycle Expression Program (CC-P). MCEP 8, primarily enriched in glandular and protein processing pathways in the endoplasmic reticulum, showed a preference for normal epithelial cells and was categorized as the Glandular Secretion Expression Program (GS-P). The spatial distributions and enrichment analyses for MCEP 5 and MCEP 8 are also shown in **Figures 1F, G**.

### 3.2 Crosstalk networks between malignant cells and immune cells mediated by differential MCEPs

**Figure 2:**
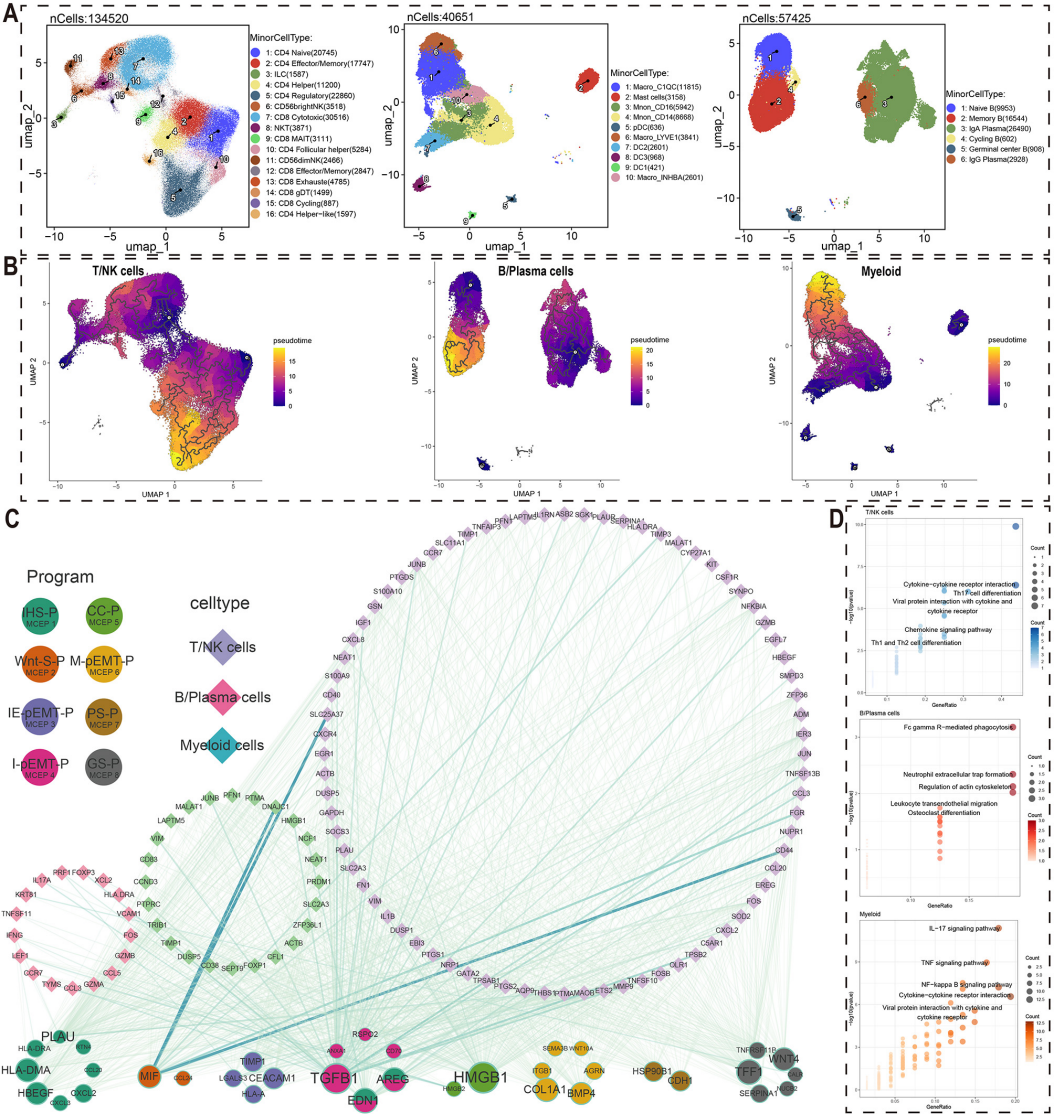
Crosstalk networks between MCEPs and immune cells. **(A)** Secondary dimensionality reduction annotation of three immune cell types (T/NK cells, B/Plasma cells, Myeloid cells). **(B)** Pseudotemporal trajectories reconstructed by Monocle3 for T/NK cells, plasma cells, and myeloid cells. **(C)** Ligand-receptor interaction network between MCEP-derived factors (circles, size scaled by target connectivity) and immune cell targets (diamonds, line width reflecting interaction strength). **(D)** Pathway enrichment analysis of target genes using hypergeometric testing, showing top five KEGG pathways per immune subset (point size: gene count; color intensity: -log10[P-value]).

To investigate the cell-cell interactions between malignant cells and immune cells, we first extracted each immune cell type (T/NK cells, B/plasma cells, and myeloid cells) from the comprehensive cell atlas for further detailed cell type annotation. T/NK cells were subdivided into 16 subpopulations, including CD4 Naive, CD4 Effector/Memory, and ILC; B/plasma cells were further categorized into 6 subpopulations, such as Naive B, Memory B, and IgA Plasma; Myeloid cells were divided into 10 subpopulations, including Macro_C1QC, Mast cells, and Mono_CD16 (**Figure 2A**, **Supplementary Figure S5-7**). Subsequently, pseudotime analysis was performed based on the secondary annotation results of each immune cell type and the stemness scores of each cell type, leading to the identification of genes associated with developmental trajectories in each immune cell population (**Figure 2B**, **Supplementary Figure S8A**).

These genes, associated with the pseudotime developmental trajectory of immune cell subsets, were used as target gene sets. For each MCEP, we selected the top 100 weighted genes in the expression programs as candidate regulators (**Figure 2C**). Among the three stress-related MCEPs, IHS-P had the highest number of regulatory factors, with HLA-DMA and PLAU affecting more target genes than other factors. In the three pEMT-related MCEPs, I-pEMT-P had the most regulatory factors, with TGFB1 having the greatest potential impact. Regulatory factors EDN1 and AREG were also abundant and shared between I-pEMT-P and IHS-P. In CC-P, HMGB1 had the most target genes, while TFF1 and WNT4 were more prominent in GS-P.

Regarding immune cell responses to MCEP crosstalk, TGFB1 and CALR were the main regulatory factors influencing T/NK cells, with TGFB1 originating from I-pEMT-P and CALR from IHS-P (**Figure 2C**, **Supplementary Figure S9A**, **Supplementary Figure S10A**). Notable downstream target genes of TGFB1 in T/NK cells included CCL3, FOXP3, and GZMB. For B/plasma cells, EDN1 and TGFB1 were the main regulatory factors, with EDN1 shared between IHS-P and I-pEMT-P (**Figure 2C**, **Supplementary Figure S9B**, **Supplementary Figure S10B**). Potential target genes of EDN1 in B cells included NCF1, PTPRC, and SLC2A3, while TGFB1 target genes included TIMP1, VIM, and CD38. In myeloid cells, the primary regulatory factors were TGFB1 and ANXA1, with ANXA1 originating from I-pEMT-P (**Figure 2C**, **Supplementary Figure S9C**, **Supplementary Figure S10C**). Potential target genes of TGFB1 in myeloid cells included ASB2, IGF1, and MMP9.

KEGG pathway enrichment analysis of the potential target genes in these immune cell subsets revealed significant biological insights (**Figure 2D**). The target genes of T/NK cells regulated by malignant cells were enriched in pathways such as Cytokine−cytokine receptor interaction, Th17 cell differentiation, and Chemokine signaling pathway, indicating a key role of cytokine networks in anti-tumor immune responses. The potential target genes of B/plasma cells were enriched in pathways such as Fc gamma R−mediated phagocytosis and Leukocyte transendothelial migration, suggesting their role in tumor-associated immunosuppression. In myeloid cells, the target genes regulated by malignant cells were enriched in IL−17 signaling pathway and TNF signaling pathway, highlighting their involvement in immune regulation and inflammation within the tumor microenvironment. These findings provide valuable biological insights for the development of future cancer therapies.

### 3.3 Crosstalk networks between malignant cells and stromal cells mediated by differential MCEPs

**Figure 3:**
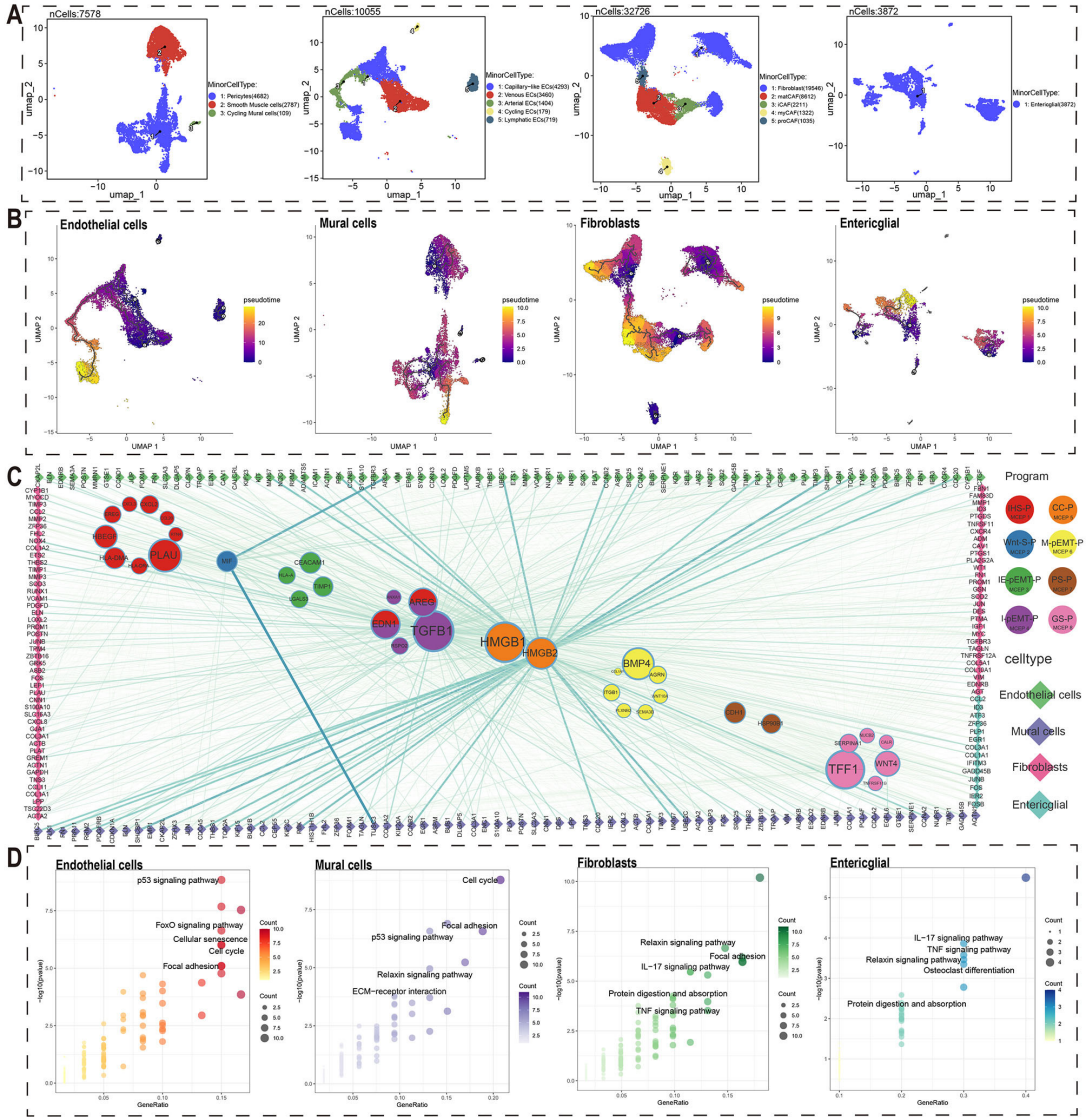
Crosstalk networks between MCEPs and stromal cells.**(A)** Secondary dimensionality reduction annotation of four stromal cell types (endothelial cells, mural cells, fibroblasts, and enteric glial cells). **(B)** Pseudo-temporal trajectory analysis of four stromal cell subtypes (endothelial cells, mural cells, fibroblasts, and enteric glial cells) shown through UMAP visualization. Color gradient (purple to yellow) indicates developmental progression from early to late stages. **(C)** Ligand-receptor interaction network between stromal cell-derived ligands (circles) and immune cell targets (diamonds). Node size corresponds to ligand-associated target quantity, line thickness represents interaction strength. **(D)** KEGG pathway enrichment of stromal cell target genes. Top five non-disease related pathways are displayed with point size indicating gene count and color intensity showing significance level (-log10[P-value]).

To investigate the effects of malignant cells on stromal cells, we performed detailed cell type annotation and stemness analysis on four stromal cell types: endothelial cells, mural cells, fibroblasts, and enteric glial cells, using methods similar to those employed for immune cell analysis (**Figure 3A**, **Supplementary Figure S8B**, **Supplementary Figure S11-14**). By integrating detailed annotations and stemness analysis, we reconstructed the developmental trajectories of these stromal cells and identified genes associated with their development (**Figure 3B**). We used high-weight genes from each MCEP as ligands to identify potential target genes in stromal cells associated with pseudotime trajectories, constructing a crosstalk network between malignant and stromal cells (**Figure 3C**).

Regarding regulatory factors in MCEPs affecting stromal cells, IHS-P had the highest number of potential regulatory factors, with PLAU affecting the most target genes. In Wnt-S-P, MIF was the only potential regulatory factor, while HSP90B1 and CDH1 were found in PS-P. Among the pEMT-related MCEPs, M-pEMT-P had more potential regulatory factors than the others, with BMP4 having the most target genes. I-pEMT-P’s top regulatory factor was TGFB1, with AREG and EDN1 also shared with IHS-P. CC-P had two regulatory factors, HMGB1 and HMGB2, with HMGB1 affecting more target genes, although HMGB2 exhibited stronger interactions with certain stromal targets. TFF1 was the top regulatory factor in GS-P.

From a stromal cell perspective, the key regulatory factors for endothelial cells were HMGB2, TGFB1, and EDN1. HMGB2 target genes, associated with proliferative endothelial cells, included ASPM, AURKB, and BIRC5 (**Figure 3C**, **Supplementary Figure S15A**, **Supplementary Figure S16A**). TGFB1 and EDN1 target genes, including CTGF, EDN1, IGF1, and CALCRL, are mainly involved in angiogenesis. For mural cells, HMGB2, TGFB1, and EDN1 were the main regulatory factors, with HMGB2 targets such as FOXM1, KIF20A, and KIF2C, expressed in proliferative mural cells. TGFB1 and EDN1 targets included CDKN1A, CNN1, COL1A1, and EDNRB, contributing to cell proliferation and stromal stability (**Figure 3C**, **Supplementary Figure S15B**, **Supplementary Figure S16B**). In fibroblasts, HMGB2, TGFB1, and EDN1 were also key regulatory factors, with TGFB1 target genes including NOX4, THBS2, and DES (**Figure 3C**, **Supplementary Figure S15C**, **Supplementary Figure S16C**). Enteric glial cells had fewer potential crosstalk genes, with top regulatory factors ANXA1, TIMP1, and HLA-A, and target genes such as COL1A1 and COL3A1, which may support tumor structure and growth (**Figure 3C**, **Supplementary Figure S15D**, **Supplementary Figure S16D**). Overall, the primary regulatory factors influencing stromal cell crosstalk were HMGB2, TGFB1, and EDN1, with HMGB2 regulating cell cycle-related targets.

Additionally, KEGG pathway enrichment analysis of potential crosstalk target genes for each stromal cell type revealed significant biological insights (**Figure 3D**). Endothelial cell targets were enriched in pathways such as the p53 signaling pathway, FoxO signaling pathway, Cellular Senescence, and Cell Cycle, suggesting their adaptability in the tumor microenvironment. Mural cell targets were enriched in the Cell Cycle, p53 signaling pathway, Focal Adhesion, Relaxin signaling pathway, and ECM-receptor interaction, emphasizing their roles in cell proliferation and matrix remodeling. Fibroblast targets were enriched in the Relaxin signaling pathway, Focal Adhesion, IL-17 signaling pathway, Protein Digestion and Absorption, and TNF signaling pathway, reflecting their dual role in immune regulation and matrix homeostasis. Enteric glial cell targets were enriched in IL-17 signaling, TNF signaling, Relaxin signaling, Osteoclast differentiation, and Protein Digestion and Absorption pathways, indicating their role in immune function and matrix support in the gut microenvironment.

### 3.4 MCEPs validation in CRC progression and development of MCEPs-related prognostic model

**Figure 4:**
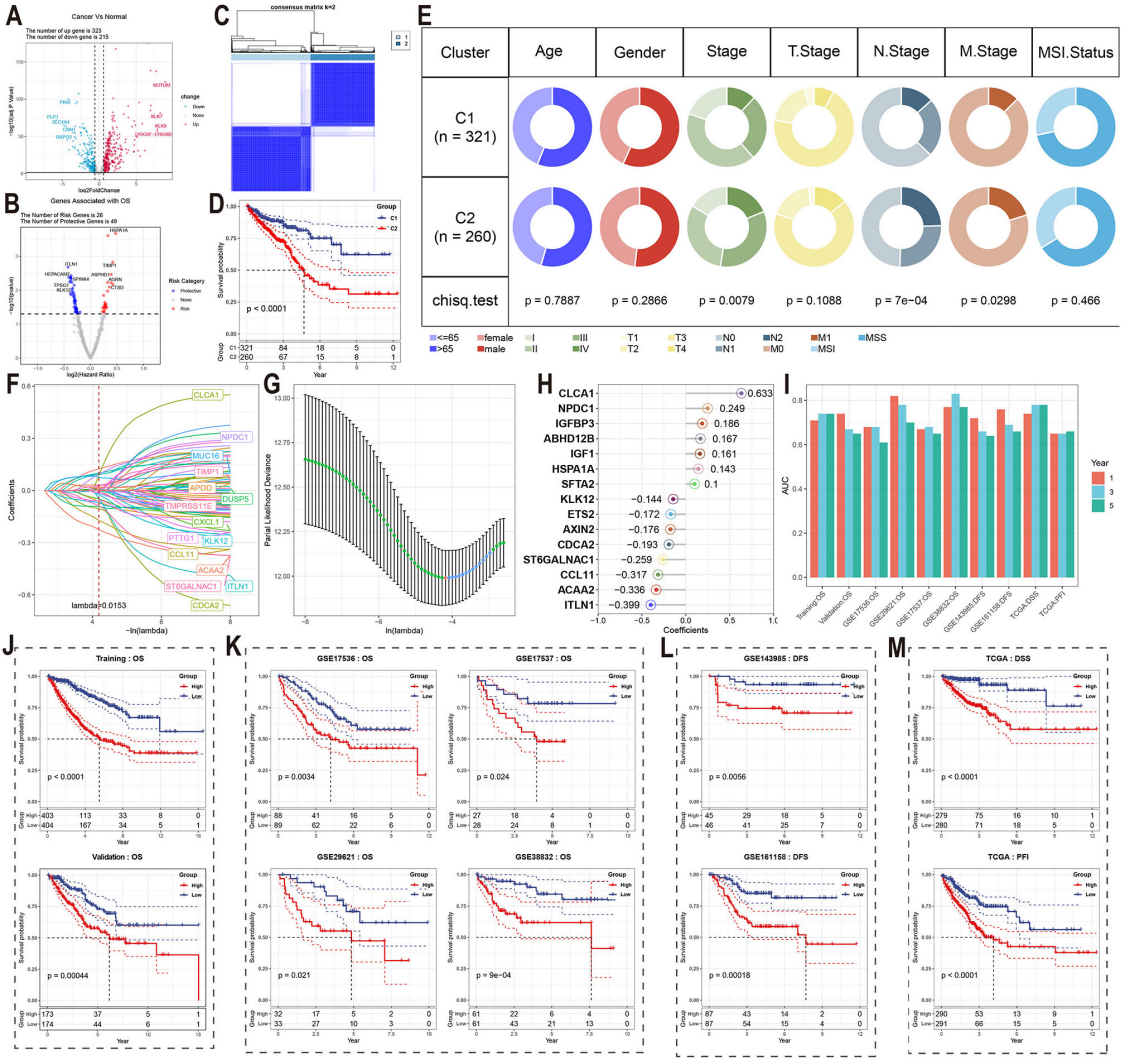
Validation of MCEPs in CRC progression and development of MCEPs-related prognostic models. **(A)** Volcano plot of differentially expressed genes (|FC| > 1.5, adjusted p < 0.05). **(B)** Univariate Cox survival analysis of prognostic genes (HR>1, red: risk factors; HR<1, blue: protective factors; p<0.05). **(C)** Consensus clustering matrix for two molecular subtypes. **(D)** Kaplan-Meier survival comparison between subtypes. **(E)** Clinical feature distribution across subtypes (χ² test). **(F)** LASSO coefficient profiles of candidate genes. **(G)** Optimal λ selection through 10-fold cross-validation (minimum deviance criterion). **(H)** Final model features with corresponding regression coefficients. **(I)** AUC values of the prognostic model across different datasets and prognostic indicators at 1-, 3-, and 5-year time points. **(J-M)** Kaplan-Meier survival curves stratified by median risk score in: **(J)** Training and internal validation sets; **(K)** External independent validation set; **(L)** DFS-specific dataset; **(M)** TCGA cohort with distinct survival endpoints.

We conducted a validation study using the TCGA CRC cohort to explore the relationship between the 8 MCEPs and CRC progression. First, we merged two gene sets: 1) the top 100 weighted genes from each MCEP module, and 2) predicted target genes from the MCEP-immune/stromal cell interaction network. Differential expression analysis was then performed comparing tumor versus normal tissues. This analysis identified 323 upregulated genes and 215 downregulated genes (**Figure 4A**).

To validate the relationship between these MCEPs and CRC onset and progression, we conducted univariate Cox regression analysis and identified 75 differentially expressed genes (DEGs) associated with survival, including 26 risk genes and 49 protective genes (**Figure 4B**). Clustering analysis based on these genes divided the TCGA cohort into two subtypes (**Figure 4C**, **Supplementary Figure S17**). Survival analysis revealed significant differences between the subtypes, with patients in subtype C1 showing significantly higher survival rates compared to those in subtype C2 (**Figure 4D**). Chi-square tests indicated significant differences in tumor stage, lymph node metastasis, and distant metastasis, suggesting that tumors in the C2 subtype progressed more rapidly and were more prone to metastasis compared to those in the C1 subtype (**Figure 4E**).Genomic analysis revealed that the most frequently mutated genes in subtype C1 were APC (70%), KRAS (48%), and TP53 (47%) (**Supplementary Figure S18A**), while in subtype C2, the most frequently mutated genes were APC (81%), TP53 (74%), and TTN (44%) (**Supplementary Figure S18B**).

A prognostic model for assessing CRC patient survival was developed using the identified genes. LASSO regression analysis was performed to reduce the feature set from the 75 survival-related DEGs identified in the previous study to 29 genes at the minimum λ value (λ = 0.0153), including genes such as CLCA1, NPDC1, and MUC16 (**Figures 4F, G**). A backward stepwise Cox regression method was then applied to further reduce the feature set to 15 genes, with the regression coefficients visualized in a lollipop plot. The combination of LASSO and backward stepwise Cox regression methods enabled the identification of the most robust prognostic markers, minimizing overfitting while ensuring the model’s predictive accuracy. Thus, these 15 genes were selected to establish the final prognostic model. Seven features had positive coefficients, with CLCA1 having the largest coefficient, while eight features had negative coefficients, with ITLN1 showing the largest absolute coefficient (**Figure 4H**).

In the training set, internal testing set, and external independent validation set, samples were divided into high-risk and low-risk groups based on the median risk score for each dataset. Significant survival differences were observed between the two groups (**Figures 4J, K**). In the training set, the AUC values for 1-year, 3-year, and 5-year survival were all greater than 0.7; in the internal testing set, the AUC value for 1-year survival was greater than 0.7, while those for 3-year and 5-year survival were above 0.65 (**Figure 4I**). The model also demonstrated excellent predictive performance in the independent validation set, with only GSE17536 showing a 5-year survival AUC value lower than 0.65. For all other datasets, the AUC values for 1-year, 3-year, and 5-year survival were all greater than 0.65. Notably, the GSE29621 dataset showed AUC values for 1-year, 3-year, and 5-year survival above 0.7, and the GSE38832 dataset exhibited even higher AUC values for all three survival endpoints, with values exceeding 0.75 (**Figure 4I**).

**Figure 5:**
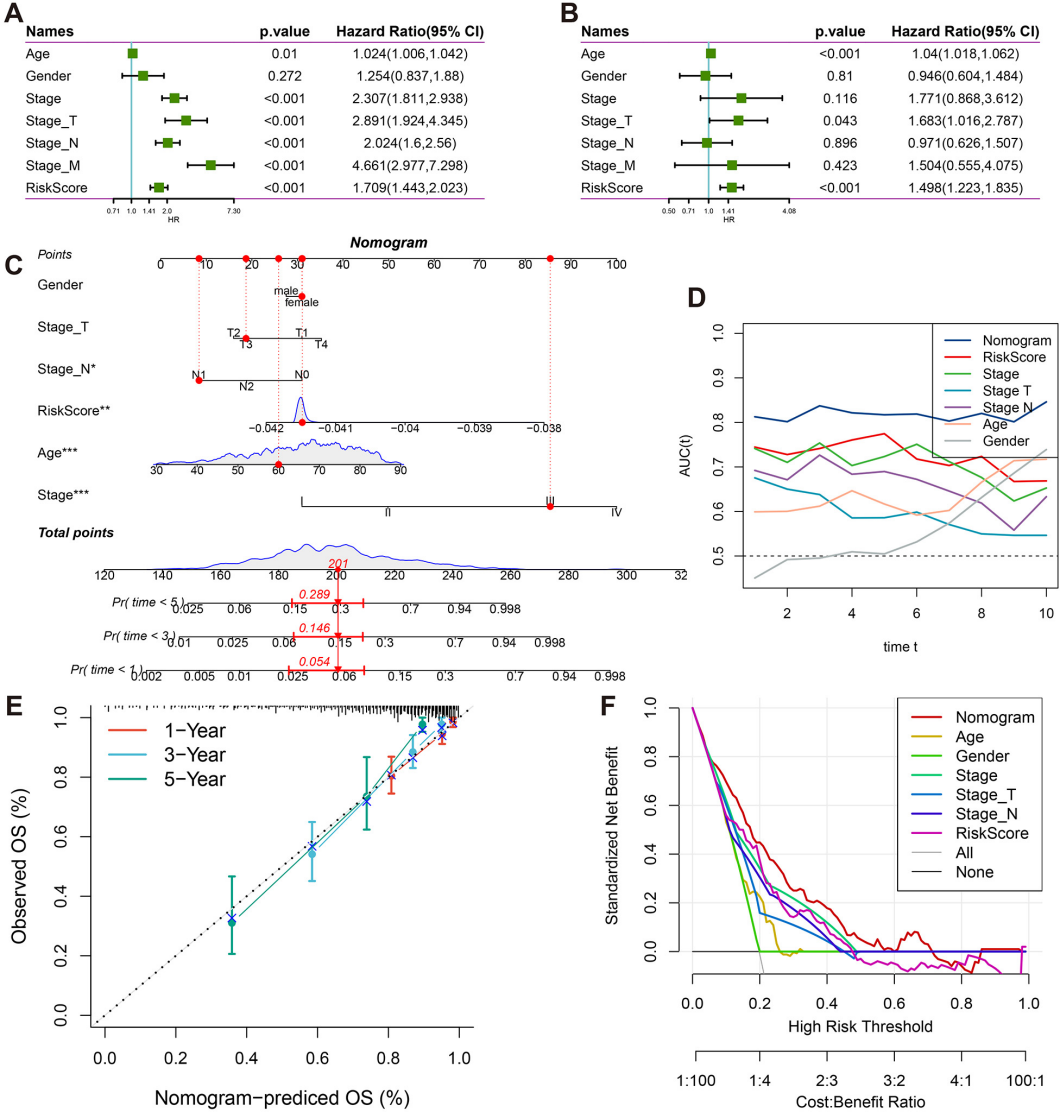
Clinical prognostic value and nomogram construction. **(A)** Forest plots of univariate Cox regression analyses for RiskScore and clinicopathological parameters (gender, age, tumor stage). **(B)** Forest plots of multivariate Cox regression analyses for RiskScore and clinicopathological parameters (gender, age, tumor stage). **(C)** Clinical nomogram integrating T/N staging, tumor stage, age, gender, and RiskScore. **(D)** Time-dependent ROC analysis (1-10 years) for nomogram performance. **(E)** Calibration curves comparing predicted vs observed survival probabilities at 1/3/5 years. **(F)** Decision curve analysis evaluating clinical utility across threshold probabilities.

To further validate the prognostic prediction capability of this model, we assessed its ability to predict disease-free survival (DFS) in the GSE143985 and GSE161158 datasets. Samples were divided into risk groups based on the median predicted risk score, and significant differences in DFS were observed between the groups (**Figure 4L**). In GSE143985, the AUC values for 1-year and 3-year DFS were above 0.65, with the 5-year DFS AUC value approaching 0.65. In GSE161158, the corresponding AUC values for DFS were above 0.65 (**Figure 4I**). The model was further validated in the TCGA cohort for disease-specific survival (DSS), progression-free interval (PFI), and disease-free interval (DFI), showing excellent predictive performance for DSS and PFI, with significant differences in median survival times (**Figure 4M**). For DSS, the AUC values for 1-year, 3-year, and 5-year survival were all above 0.7, and for PFI, the AUC values were above 0.65 (**Figure 4I**). Notably, the model consistently achieved stable predictive accuracy across six independent validation cohorts (GSE17536, GSE17537, GSE29621, GSE38832, GSE143985, and GSE161158) and multiple clinical endpoints (OS, DFS, DSS, PFI), highlighting its strong generalizability to diverse patient populations and survival outcomes.

### 3.5 Multidimensional biological interpretation of the prognostic model

**Figure 6:**
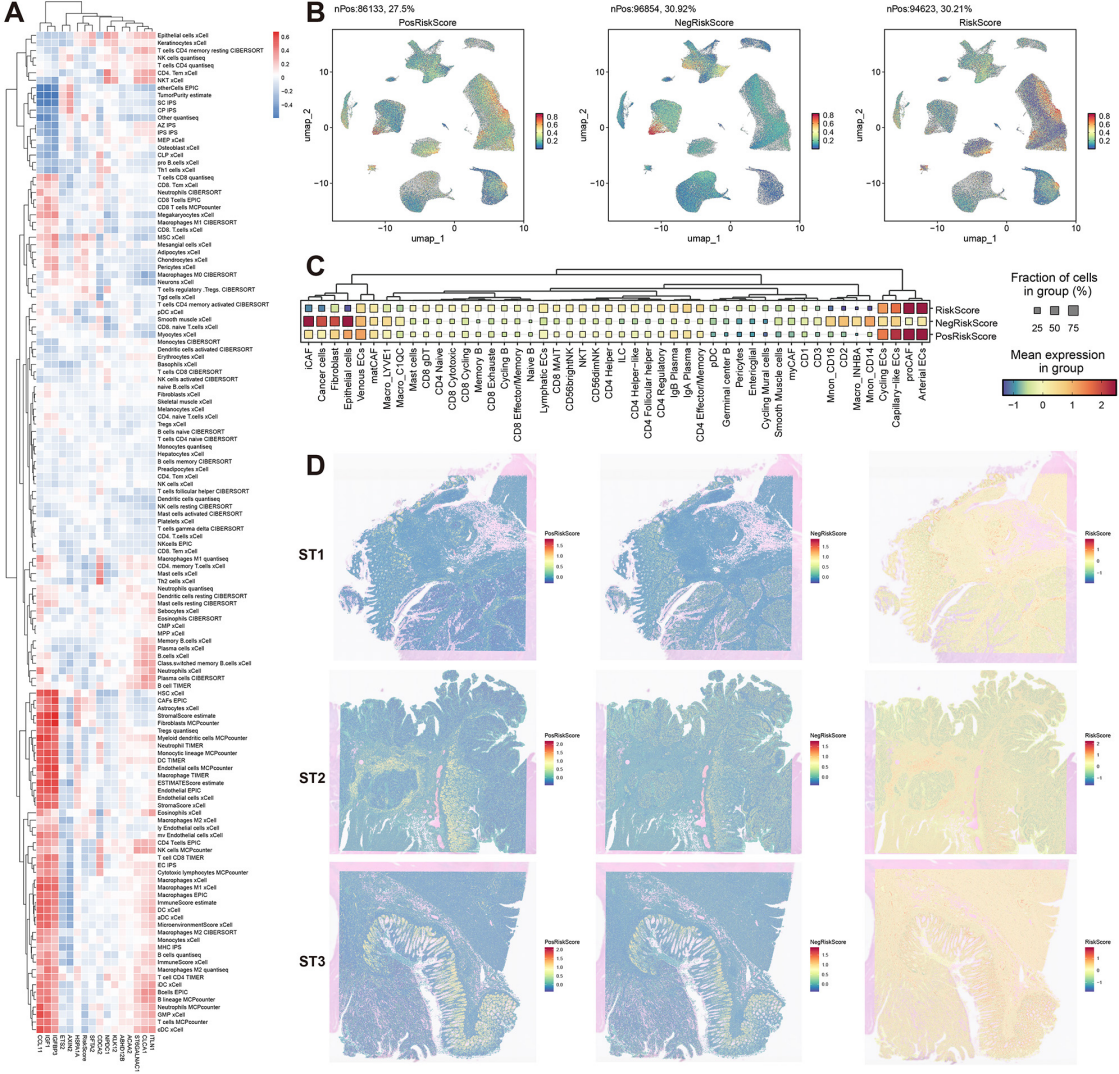
Multidimensional biological interpretation of prognostic signatures. **(A)** Spearman correlation heatmap between model genes and deconvoluted immune cell populations (red: positive, blue: negative). **(B)** Single-cell UMAP projections visualizing risk-associated signatures: PosRiskScore (positive coefficient genes), NegRiskScore (negative coefficient genes), and composite RiskScore. **(C)** Dot plot displaying cell type-specific enrichment of risk signatures (dot size: scoring cell proportion; color intensity: score magnitude). **(D)** Spatial distribution patterns of risk signatures across three representative specimens (ST1-ST3).

To gain further insights into the biological underpinnings of the prognostic model, the cellular abundance of various cell types in the TCGA cohort was first calculated using deconvolution methods. Next, the correlation between each gene in the prognostic model and the cell scores was computed, revealing that CCL11, IGF1, and IGFBP3 were significantly correlated with multiple cell types. Specifically, these genes were positively correlated with cancer-associated fibroblasts, stromal score, and Tregs, while negatively correlated with tumor purity (**Figure 6A**).

The model was then further dissected at the single-cell level. Using genes with positive coefficients, a PosRiskScore for each cell was calculated, and similarly, a NegRiskScore was calculated using genes with negative coefficients. The total RiskScore for each cell was derived by computing the difference between PosRiskScore and NegRiskScore. The distribution of these scores was first visualized, and distinct distribution patterns for PosRiskScore and NegRiskScore were observed (**Figure 6B**). Specifically, PosRiskScore was found to be higher in endothelial cells and pre-cancer-associated fibroblasts (preCAFs), potentially linked to angiogenesis and epithelial-mesenchymal transition. In contrast, NegRiskScore was elevated in iCAFs, epithelial cells, normal fibroblasts, and myeloid immune cells, with NegRiskScore correlating with iCAFs and myeloid immune cells, which might reflect the inflammatory characteristics of the tumor microenvironment. Higher scores in epithelial cells were also observed, which could be indicative of a more epithelial-like phenotype associated with partial EMT processes (**Figure 6C**).

Furthermore, the analysis was extended to the spatial transcriptomics level. It was shown that PosRiskScore was predominantly localized in the stromal regions of malignant cell areas, while NegRiskScore was mainly concentrated in the epithelial regions. Consequently, the final RiskScore had the lowest score in the epithelial areas and the highest score in the stromal regions, with similar distribution patterns observed across three samples (**Figure 6D**). Overall, the positive coefficient features in the prognostic model were likely to represent higher levels of mesenchymal traits associated with pEMT, while the negative coefficient features were likely linked to a more inflammatory microenvironment and epithelial characteristics of pEMT. Thus, the final RiskScore reflected the relative balance between epithelial-mesenchymal features and the degree of inflammation in the tumor microenvironment, offering valuable insights into patient prognosis.

### 3.6 Integration of risk score and clinical features to construct a nomogram for prognosis prediction

To enhance the prognostic accuracy and clinical applicability of the model, univariate Cox regression analysis was performed on age, gender, clinical stage, Stage_T, Stage_N, Stage_M, and RiskScore (**Figure 5A**). Significant survival risk factors were identified for all features except gender. In multivariate Cox regression analysis, age, Stage_T, and RiskScore were found to be independently associated with survival, confirming RiskScore as an independent prognostic factor (**Figure 5B**).

A nomogram was subsequently constructed, incorporating age, gender, clinical stage, Stage_T, Stage_N, and RiskScore (**Figure 5C**). It was demonstrated that the nomogram improved clinical decision-making compared to traditional staging systems through three key mechanisms: First, continuous risk quantification allowed for more precise stratification of patient outcomes than categorical staging classifications. Second, the multidimensional integration of molecular risk scores with clinicopathological parameters provided complementary prognostic information that surpassed the limitations of anatomical staging alone. Third, the dynamic estimation of survival probability for specific timepoints (1-10 years) facilitated personalized follow-up planning and therapeutic decision-making. Stage_M was excluded from the analysis due to collinearity with overall stage.

Excellent predictive performance was demonstrated by the nomogram, with AUC values exceeding 0.8 for survival predictions at 1, 3, 5, and 10 years (**Figure 5D**). Strong agreement between predicted and actual survival probabilities was observed in calibration curves for 1, 3, and 5 years (**Figure 5E**). Clinical decision curve analysis revealed that the nomogram consistently provided higher net benefits across various threshold probabilities when compared to both individual clinical parameters and traditional staging systems (**Figure 5F**). The enhanced clinical utility of the nomogram was attributed to its ability to synthesize molecular biomarkers with conventional staging data, addressing the heterogeneity within traditional stage categories and enabling more individualized risk assessment. These findings collectively validated the effectiveness and clinical applicability of the proposed model.

### 3.7 Potential drug therapeutic targets based on MCEPs

**Figure 7:**
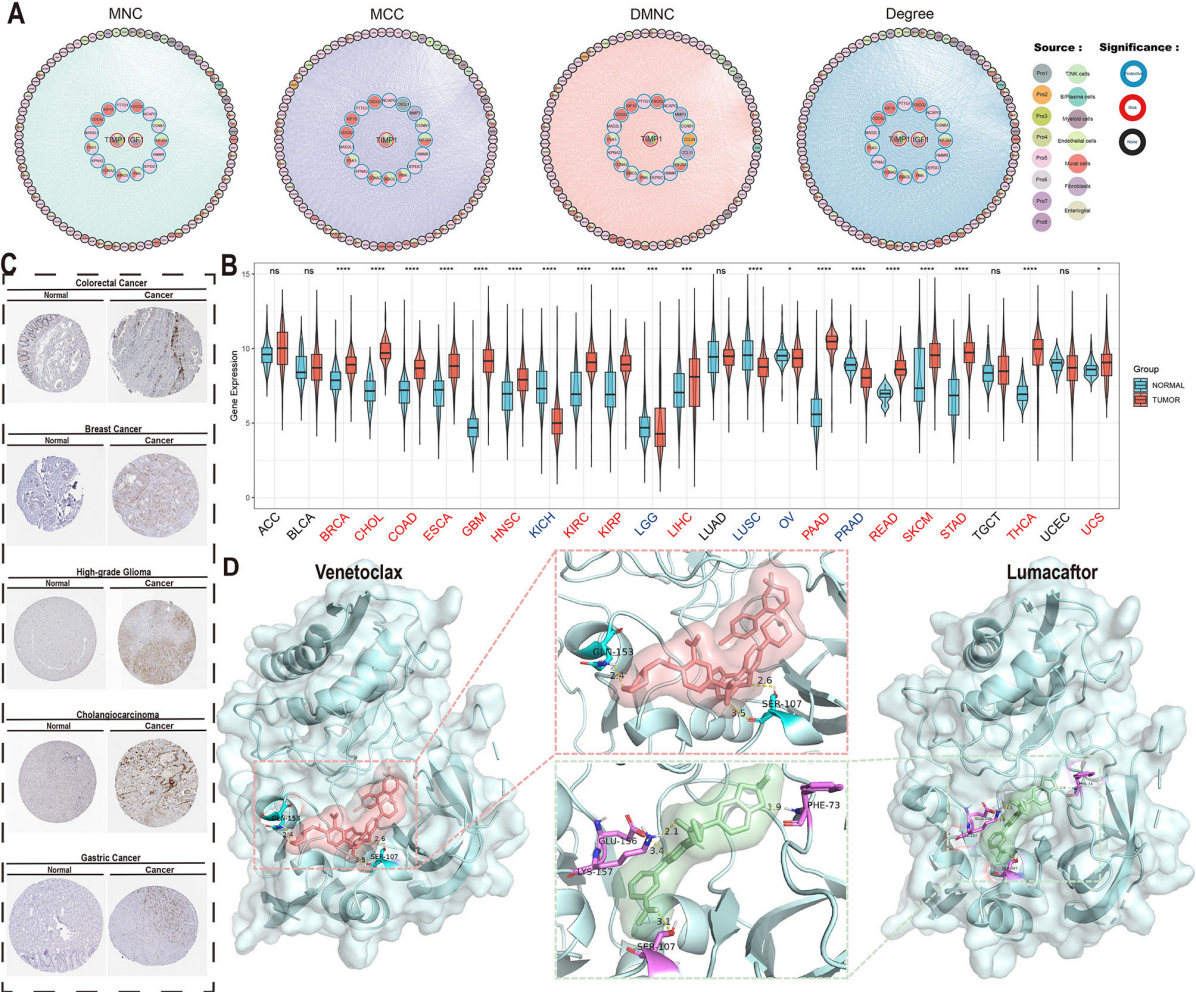
Core determinant identification and therapeutic exploration. **(A)** Protein-protein interaction (PPI) network of top 100 survival-associated genes identified through MNC/MCC/DMNC/Degree algorithms (border color: survival association; fill color: algorithm source). **(B)** TIMP1 differential expression across TCGA tumor types versus normal tissues (ns, P > 0.05; *, P < 0.05; **, P < 0.01; ***, P < 0.001; ****, P < 0.0001). **(C)** TIMP1 immunohistochemical validation in multiple carcinomas and paired normal tissues. **(D)** Molecular docking of TIMP1 (PDB:3V96) with Lumacaftor (ΔG = -12.13 kcal/mol) and Umbralisib (ΔG = -11.80 kcal/mol), showing binding pocket configurations.

To identify actionable therapeutic targets in CRC, we systematically analyzed 538 DEGs through PPI network construction. Four distinct topological algorithms (MNC, MCC, DMNC, Degree) were employed to prioritize the top 100 hub genes from the PPI network. Subsequent survival impact analysis revealed that TIMP1 and IGF1 emerged as prognostic risk genes among these hub genes. Notably, TIMP1 exhibited consistent identification across all four algorithms, whereas IGF1 was only captured by MNC and Degree algorithms (**Figure 7A**). Based on its algorithm-independent prioritization and significant association with poor prognosis, TIMP1 was selected as the principal therapeutic target for further investigation.

Pan-cancer expression profiling demonstrated significant TIMP1 upregulation in 15 malignancies (including colorectal adenocarcinoma [COAD], breast invasive carcinoma [BRCA], and cholangiocarcinoma [CHOL] as representative examples), while downregulation was observed in 10 cancer types (exemplified by kidney chromophobe [KICH] and lung squamous cell carcinoma [LUSC]) with no significant alterations detected in other malignancies (**Figure 7B**). Immunohistochemical validation via the Human Protein Atlas confirmed elevated TIMP1 protein levels in CRC, breast cancer, glioma, hepatocellular carcinoma, and gastric adenocarcinoma (**Figure 7C**), underscoring its pan-cancer relevance.

Virtual screening of 2,000 bioactive compounds against the TIMP1 structure identified Venetoclax (ΔG = -12.236 kcal/mol) and Lumacaftor (ΔG = -12.129 kcal/mol) as top candidates with superior binding affinities (**Figure 7D**). Molecular docking simulations predicted stable interactions between these compounds and key TIMP1 functional domains.

These findings computationally nominate TIMP1 as a multi-cancer therapeutic target, with the identified small-molecule inhibitors warranting preclinical evaluation for targeted therapy development in CRC and other TIMP1-driven malignancies.

## 4 Discussion

In this study, we re-examined the biological characteristics of CRC by leveraging prior research on malignant cell transcriptional signatures and identified eight major MCEPs (32). These programs encompass three stress-related categories (hypoxia-inflammation, Wnt-related, and proliferation), three EMT subtypes (inflammatory epithelial, intermediate, and mesenchymal), one cell cycle category, and one glandular secretion category. Each program is critically linked to functional roles in regulating malignant cell proliferation, migration, drug resistance, metastasis, and patient prognosis (33–36). Traditional molecular subtyping approaches, such as those based on hypoxic metabolism, cellular senescence, or microenvironmental cell markers (37–39), often oversimplify tumor heterogeneity. Solid tumors are multifactorial systems, and reliance on binary phenotypic classifications risks underestimating inter-individual variability and obscuring underlying biological processes, thereby limiting the molecular interpretability of subtypes.

To address this, we employed a programmatic state-based framework to characterize CRC gene expression, accounting for potential confounders and mutual exclusivity between states. Importantly, we emphasized continuity within each state rather than discrete isolation. For instance, malignant cell partial EMT was defined as a tripartite continuum (mesenchymal, intermediate, and epithelial), aligning with the evolving concept of “epithelial-mesenchymal plasticity” endorsed by the International EMT Association (40). The tumor microenvironment, a complex ecosystem sculpted predominantly by malignant cells, has historically been analyzed by grouping tumor cells homogeneously or partitioning them into static clusters. In contrast, our crosstalk analysis originated from malignant cell expression programs, enabling simultaneous exploration of heterogeneity in both malignant and stromal/immune compartments.

In our analysis of the eight MCEPs, we identified critical regulators with potential crosstalk interactions in immune/stromal compartments, including TGFβ1 and HMGB1. Functional annotation of downstream target genes in immune/stromal cells revealed biological roles consistent with established mechanisms. Specifically, TGFβ1 signaling dysregulation plays a pivotal role in colorectal carcinogenesis by governing cell growth, differentiation, migration, and apoptosis (41–43). Pathological overexpression of TGFβ1 drives epithelial-mesenchymal transition, extracellular matrix remodeling, and cancer-associated fibroblast activation (44–46). Notably, TGFβ1 emerged as a key regulator in the I-pEMT-P program, targeting immune cell genes including FOXP3, CD38, and MMP9—established mediators of immune evasion and immunosuppressive TME remodeling (47–49). In stromal compartments, TGFβ1 may further facilitate CAF transformation and immunosuppressive functions through NOX4-mediated pathways (50).Meanwhile, nuclear HMGB1 functions as a chromatin-binding factor regulating nucleosome organization, transcriptional control, and genomic stability, whereas extracellular HMGB1 modulates cell differentiation, metastatic dissemination, and apoptosis (51). Concurrently, HMGB2 within the CC-P program demonstrated regulatory effects on mesenchymal-like cells, modulating pro-angiogenic genes such as AURKB, BIRC5, and FOXM1 that coordinate endothelial and vascular smooth muscle cell proliferation (52–54). This integrated regulatory network analysis reveals how malignant cell-derived signals orchestrate multicellular ecosystem dynamics through conserved molecular pathways, providing mechanistic insights into TME reprogramming during CRC progression.

CRC prognosis remains challenging due to pronounced tumor heterogeneity. Existing prognostic models, often anchored to singular features (e.g., immune, EMT, or metabolic signatures), provide incomplete assessments. Our integrative model, combining immune and stromal features, offers enhanced biological interpretability. Risk stratification revealed that high-risk scores correlate with mesenchymal-like, immunosuppressive TMEs enriched in CAFs, Tregs, and inflammatory markers. Conversely, low-risk scores associate with epithelial-like phenotypes marked by partial EMT, reduced stromal activation, and preserved epithelial integrity. The model incorporates 15 genes, with CLCA1 and ITLN1 exhibiting the strongest prognostic weights. CLCA1, a tumor suppressor, inhibits CRC progression by suppressing Wnt/β-catenin signaling and EMT, consistent with its reduced expression in advanced tumors and inverse correlation with metastasis (55). ITLN1, conversely, antagonizes tumor neovascularization and MDSC accumulation via IL-17D/CXCL2 axis modulation, thereby reshaping the immunosuppressive TME—a mechanism aligning with its prognostic significance in both CRC and ovarian cancer (56, 57). Additional contributors, such as IGFBP3 and ACAA2, further underscore the multifactorial nature of CRC heterogeneity. Elevated IGFBP3, driven by genetic predisposition, may enhance CRC risk through IGF1-mediated mitogenic signaling, as supported by Mendelian randomization analyses (58). ACAA2, a fatty acid metabolism enzyme, inversely correlates with cetuximab resistance, particularly in KRAS-mutant CRC, suggesting its role in metabolic adaptation and therapy response regulation (59). This framework bridges molecular mechanisms to clinical outcomes, providing biological interpretability to the prognostic model.

As an independent prognostic factor, our model achieved an AUC >0.8 for 10-year outcome prediction when combined with clinical variables. Integration with TNM staging via a nomogram improves CRC management by enabling dynamic survival probability estimation (1–10 years), optimizing adjuvant therapy selection, surveillance intervals, and resource allocation.

PPI network analysis identified TIMP1 as a hub gene within the I-pEMT-P program. TIMP1, a matrix metalloproteinase inhibitor, exhibits context-dependent roles in cancer. In brain metastases, astrocyte-derived TIMP1 suppresses CD8^+^ T cell activity (60), while in pancreatic cancer, TIMP1-CD63-ERK signaling drives neutrophil extracellular trap formation and tumor progression (61). In CRC, TIMP1 correlates with tumor cell proliferation, invasion, and poor prognosis (62). Our data suggest that the I-pEMT-P program may remodel the stromal niche via TIMP1, influencing tumor progression and clinical outcomes.

### 4.1 Limitations and future directions

Despite the significant findings, this study has some limitations. Although single-cell data from over 100 samples were analyzed, the lack of clinical annotations, such as tumor stage, survival time, and survival status, restricted our ability to directly correlate expression programs with tumor progression and patient outcomes. Therefore, we relied on bulk RNA-seq datasets, which included complete clinical information. Additionally, while computational predictions identified key regulators, such as TGFβ1 and HMGB2, in stromal/immune modulation, their mechanistic roles remain unvalidated experimentally. Future studies should employ co-culture models or *in vivo* systems to confirm these interactions.

## 5 Conclusion

This study identified eight distinct MCEPs that characterize the transcriptional states of CRC malignant cells. We constructed interaction networks between these MCEPs and immune or stromal cells, which led to the development of a prognostic model consisting of 15 genes. Furthermore, TIMP1 was identified as a key gene, and two potential drugs, Venetoclax and Lumacaftor, were highlighted for targeted therapeutic strategies. In summary, this study provides new insights and references for CRC heterogeneity and prognostic therapy.

## Data Availability

Publicly available datasets were analyzed in this study. Gene Expression Omnibus (GEO): Datasets such as GSE166555, GSE200997, GSE39582, GSE17536, GSE17537, GSE29621, GSE38832, GSE143985, and GSE161158 are available in the GEO database. Synapse Database: The dataset with accession number syn26844071 is hosted on Synapse.10x Genomics: The Visium HD colorectal cancer sequencing datasets can be accessed on the 10x Genomics website. The Cancer Genome Atlas (TCGA) and UCSC Xena: TCGA provides bulk RNA-seq data, SNV data, pan-cancer clinical data, and pan-cancer gene expression data. The Human Protein Atlas: Immunohistochemistry images are available from The Human Protein Atlas.
