# Supplementary material for: Single-cell and spatial transcriptomic analysis reveals tumor cell heterogeneity and underlying molecular program in colorectal cancer
Source: Front Immunol. 2025 Mar 12;16:1556386. doi: 10.3389/fimmu.2025.1556386 (PMC11936967; doi:10.3389/fimmu.2025.1556386)
Supplement: Supplementary file 1 [file DataSheet1.docx]

**Supplementary Material**

**Single-Cell and Spatial Transcriptomic Analysis Reveals Tumor Cell Heterogeneity and Underlying Molecular Program in Colorectal Cancer**

Teng Wang^1^, Zhaoming Chen^1^, Wang Wang^2,3^, Heng Wang^1^, Shenglong Li^1*^

*^1^Department of Bioinformatics, School of Basic Medical Sciences, Chongqing Medical University, Chongqing 400010, China*

*^2^Department of Immunology, School of Basic Medical Sciences, Chongqing Medical University, Chongqing 400010, China*

*^3^Chongqing Key Laboratory of Tumor Immune Regulation and Immune Intervention, Chongqing Medical University, Chongqing 400010, China*

| **TableS1 Top 100 high-weight genes of malignant cell expression programs** | | | | | | | |
| --- | --- | --- | --- | --- | --- | --- | --- |
| MCEP1 | MCEP2 | MCEP3 | MCEP4 | MCEP5 | MCEP6 | MCEP7 | MCEP8 |
| TMSB4X | TMEM176A | S100P | PLA2G16 | HMGB2 | CRIP1 | HSP90AB1 | FCGBP |
| SLC25A6 | PCBP2 | TSPAN1 | MAL2 | TUBA1B | CDA | HSPD1 | SPINK4 |
| PHLDA2 | RNF43 | CYSTM1 | KRT7 | H2AFZ | KLK8 | HSP90AA1 | REG4 |
| CYCS | MFAP2 | FXYD3 | CRIP2 | PTTG1 | PSCA | HSPA8 | KLK1 |
| ODC1 | ATP6V0C | ISG20 | NDUFB9 | BIRC5 | TM4SF4 | HSPA5 | MUC2 |
| SFN | JUN | MYL12A | ACAA2 | CDC20 | S100A4 | MTDH | SH3BGRL3 |
| KLF6 | ATP5MC2 | CTSE | NPDC1 | MAD2L1 | PADI1 | HSPE1 | TFF3 |
| EPHA2 | GABARAP | IFI27 | EIF3H | NUSAP1 | FAM83A | MTPN | TFF1 |
| TNFRSF12A | ALDOA | MYL12B | AQP5 | UBE2C | A4GALT | HSP90B1 | FAM177B |
| RAB5IF | NME2 | CLDN7 | BRI3 | CCNB1 | C19orf33 | MTCH1 | AGR2 |
| CXCL3 | IFITM1 | TM4SF5 | GALNT1 | TPX2 | TNNT1 | MTIF3 | B3GNT6 |
| DSTN | TMEM176B | TSPAN3 | MZT2A | CDKN3 | CRABP2 | HSPA4 | MUC5B |
| FOSL1 | GPX2 | ANXA2 | PPP1R14B | TOP2A | UNC93B1 | MTCH2 | CREB3L1 |
| SNRPB | NGRN | DPP4 | PABPC1 | SMC4 | KLK11 | HSPA9 | ITLN1 |
| EIF6 | COL9A3 | SDCBP2 | NPC2 | HMGB1 | COL6A1 | DSP | REP15 |
| SDC4 | SLC39A5 | TMEM45B | SRSF9 | CKS1B | S100A13 | MTIF2 | LGALS4 |
| RAC1 | NOTUM | S100A6 | NXPH4 | UBE2T | ITGB4 | TMEM238 | SPDEF |
| PIM3 | ITPR2 | B2M | BTF3 | HMMR | MMP7 | MTX2 | ST6GALNAC1 |
| HLA-DRA | FOS | SLPI | PLAC8 | CCNA2 | KRT16 | HSPH1 | FMOD |
| PLAUR | PTPRO | COX7A2 | FAM84B | CCNB2 | COL3A1 | MTHFD2 | KIAA1324 |
| COMMD7 | ARF5 | PRSS3 | IER3IP1 | CDK1 | DEFB1 | PRKDC | CMIP |
| MAP2K3 | CDX2 | TMEM92 | INO80C | CENPW | CTSB | IGF2BP1 | MIA3 |
| TMEM230 | DNAJB1 | LGALS2 | TATDN1 | HMGN2 | KLK7 | PTBP3 | FRZB |
| MAFF | NKD1 | LGALS3 | ASAP1 | NUF2 | COL1A1 | PRPF40A | HEPACAM2 |
| MRPL12 | COMMD6 | ANXA10 | RNF139 | DTYMK | TRIM54 | MTUS1 | F3 |
| EREG | EEF2 | SDR16C5 | HMGA1 | ZWINT | SLC2A1 | GOLGB1 | IGLC7 |
| HBEGF | POLR1D | ANG | TACSTD2 | PBK | MUC16 | HSPA1B | MYADM |
| HLA-DPA1 | WBP1 | ZG16B | RACK1 | PLK1 | UNC5B-AS1 | XRCC5 | TSPAN13 |
| AREG | NACA | CIB1 | HEBP2 | MKI67 | CD82 | MTMR6 | AQP3 |
| HLA-DRB1 | GADD45B | HSD17B2 | RSPO2 | TK1 | SFTA2 | ROCK2 | CPS1 |
| CRLS1 | EEF1A1 | GCNT3 | ATP5F1A | GTSE1 | ITGA3 | MTREX | WNT4 |
| IER3 | PPIA | CSTB | HSD17B1 | STMN1 | COL1A2 | GNAS | S100A6 |
| CEBPA | AXIN2 | SMIM24 | TMED3 | RANBP1 | ASPHD1 | HSPB11 | LRRC26 |
| TNFAIP3 | APEX1 | ERO1A | CD70 | NEK2 | SPON2 | ATRX | SELENOM |
| HLA-DMA | VAV3 | SLC22A18 | EIF4EBP1 | AURKB | IGFBP3 | KCNQ1OT1 | C4BPB |
| PRDX5 | CYP2W1 | ITM2B | HES4 | TUBB | PLXNB2 | LAMP1 | CALR |
| DPM1 | ZFP36L1 | ASS1 | UQCRH | CENPA | AHNAK2 | HSPA1A | CAPN9 |
| FGGY | PFDN5 | HLA-B | FRMD4B | RRM2 | ANO1 | RAB11FIP1 | FOXA3 |
| TM4SF1 | QTRT1 | IFI6 | C6orf48 | KIF20B | CLIC3 | HSPB1 | RAP1GAP |
| MCL1 | PSMA6 | MALL | CAPS | CEP55 | TIMP2 | NPTX2 | BPIFA2 |
| PSMA1 | CRYL1 | SLC40A1 | RAC3 | CDCA3 | OST4 | MTF2 | MLPH |
| PSMA2 | MRPS24 | MGST3 | AGPAT2 | DLGAP5 | AFAP1L2 | GOLIM4 | FGL1 |
| SOD2 | AMACR | PKIB | GPX4 | DEPDC1 | COX6B2 | ERAP2 | GSN |
| CLDN4 | SESN1 | PLS1 | C9orf16 | CDCA5 | KRT19 | ACTR2 | ENTPD8 |
| ATF4 | ERGIC3 | SMIM22 | SLC43A3 | CKAP2 | WNT10A | CANX | CBFA2T3 |
| CXCL2 | TSC22D1 | TIMP1 | ETV1 | RAN | ECM1 | NCL | SCNN1A |
| CXCL1 | HM13 | CD55 | SNX3 | CENPE | DCBLD2 | MARCKS | C16orf54 |
| NAA10 | GNG10 | SLC6A8 | CHMP1B | TACC3 | KLK6 | LRRC75A | CPE |
| PLAU | LETMD1 | PDZK1IP1 | AREG | AURKA | XIST | MTPAP | FOXC2 |
| EIF3E | IER2 | MUC13 | ATP6V1G1 | UBE2S | MIA | PCCA | ALDH1L1 |
| HLA-DPB1 | SOX4 | S100A10 | ANXA1 | ECT2 | LRRTM1 | HSPA13 | COL16A1 |
| SNRPB2 | WDR83OS | CEACAM1 | GLO1 | KIF4A | GABRP | HSPBP1 | TCIM |
| OAZ1 | DPEP1 | SMPDL3A | YBX1 | CENPM | IGFBP6 | BPTF | ALDH1A2 |
| DUSP5 | BTG2 | SLC16A3 | TMEM123 | RACGAP1 | IGFL2-AS1 | AFDN | TOX |
| MAPRE1 | PCMTD2 | MUC1 | C9orf116 | KIF23 | CYP4F3 | TAOK1 | SERPINA1 |
| ARL14 | CCL24 | QSOX1 | NAP1L1 | PCLAF | LEMD1 | MTR | GALNT3 |
| IL2RG | PHB2 | S100A14 | PHPT1 | NUCKS1 | FAM3C | ERCC4 | AGR3 |
| NFKBIA | IGF2.1 | NQO1 | ST3GAL4 | CDCA8 | APLP2 | MTG2 | BCAS1 |
| C2 | CDKN1B | TMBIM1 | AC097478.1 | KIF2C | PROM2 | CDH1 | B3GNT7 |
| ERRFI1 | VPS36 | PTTG1IP | DSG2 | ASPM | APLP1 | NORAD | NUCB2 |
| TRIB1 | DBNDD2 | PPIC | ARPC1A | TROAP | SLC26A9 | MTX3 | SMIM14 |
| IL32 | MPP1 | HLA-A | ZNF511 | DHFR | AGRN | PAK2 | ATP2A3 |
| GADD45A | APCDD1 | APOD | NR4A3 | SMC2 | ZBED2 | CD81 | DUSP4 |
| CSRNP1 | ZKSCAN1 | S100A11 | GLRX5 | SPC25 | SLC4A11 | TGOLN2 | PDIA4 |
| BIRC3 | TSPAN6 | AL355312.4 | EIF3L | SKA2 | CARNS1 | CSNK1A1 | CLCA1 |
| PRELID3B | EIF4B | OPTN | AC025154.2 | ANLN | TNNI3 | HNRNPAB | UAP1 |
| FKBP1A | TLE2 | CDH17 | GUK1 | SGO1 | TYMP | TTC3 | RAB27B |
| LURAP1L | HMGN4 | PLSCR1 | SEC11A | MYBL2 | KRT80 | TMPRSS11E | VSIG1 |
| ZFP36 | PROX1 | VILL | NPM3 | PTMA | TRIM29 | HSPA6 | SERPINB5 |
| CCL20 | GSTK1 | GBP3 | PDCD6 | RPA3 | ABHD12B | PMEPA1 | C12orf57 |
| HLA-DQB1 | LGR5 | KRT19 | FHL2 | TYMS | NOTCH3 | SET | TMEM263 |
| PFDN4 | WASHC1 | MSLN | CSNK2B | FOXM1 | CAVIN3 | ZBTB20 | CD55 |
| LY6G6F-LY6G6D | ROMO1 | TM4SF20 | SMAD2 | ARHGAP11A | S100A11 | MTHFD1L | DNAJC10 |
| CHMP4B | CLDN15 | GLRX | RAC1 | KIF11 | ENO2 | HLA-C | PMP22 |
| CD74 | PCK1 | AGR2 | ZNF706 | OIP5 | UNC13D | ZBTB38 | TPSG1 |
| PARD6B | COA3 | IFI27L2 | EDN1 | CENPU | SDC1 | MTMR14 | AC005833.1 |
| NDUFB8 | TUBA1A | PTPRH | TMEM14B | PCNA | TRIB2 | MTO1 | SEC24D |
| NDUFC2 | TMEM9 | CD68 | TMEM14C | LMNB1 | CCDC9B | NSA2 | ASPH |
| NAA20 | PRPF6 | S100A16 | MRPL14 | CENPF | SPON1 | PIK3R1 | ASRGL1 |
| CHPT1 | MIF | TMSB10 | SNHG7 | GINS2 | ITGB1 | LRPPRC | FABP2 |
| RTN4 | IMPDH2 | TSPAN8 | CARD19 | MCM7 | NDUFB1 | MTHFD1 | SLC18A1 |
| EPCAM | DAZAP2 | RHOF | BSPRY | HNRNPA2B1 | RUNX3 | BOD1L1 | KLK12 |
| PTPN7 | C8orf59 | RHOC | HNRNPA1 | KPNA2 | BST2 | SPTBN1 | ATP1B1 |
| EDN1 | COX16 | VAMP8 | TRIB1 | CENPN | EPS8L1 | VPS35 | CRELD2 |
| EIF4A1 | EEF1B2 | SLC44A4 | PIM1 | NCAPG | MUC20-OT1 | VMP1 | GLUL |
| SLC25A33 | ZFAS1 | PRSS8 | AC005256.1 | SGO2 | SLC2A3 | TAF10 | MATN2 |
| EMP1 | MAGED2 | SPINT2 | BRK1 | RAD51AP1 | CTSD | CD2AP | AP002498.1 |
| SMIM26 | CDCA7 | FABP1 | RALBP1 | BUB1 | C4orf48 | ERCC1 | SLC4A4 |
| CEBPB | PPDPF | XRCC4 | TRIOBP | NCAPH | DUSP27 | YWHAB | RBP4 |
| C10orf99 | C17orf77 | MX1 | TPGS2 | ORC6 | NRP2 | GSPT1 | TACSTD2 |
| KLF10 | TEX45 | C19orf33 | BAG1 | KIFC1 | BMP4 | MTMR12 | HOXB2 |
| TRAF4 | MUC3A | LGALS4 | HOMER2 | TMPO | ONECUT3 | GFPT1 | AC011523.1 |
| NME1 | TM9SF2 | C4orf3 | TMEM54 | MZT1 | SEMA3B | GAS6-AS1 | TNFRSF11B |
| PSMA7 | EIF4EBP3 | DUOX2 | GADD45A | DIAPH3 | IGHG3 | MTMR2 | XBP1 |
| GJB3 | TCEAL4 | IFI35 | CXCL8 | CENPK | KRT6B | AL136454.1 | EDEM3 |
| FAM3B | ALDH1B1 | LINC01133 | MARCKSL1 | PIMREG | LAT | G3BP1 | QSOX1 |
| C6orf132 | PRR15L | COX7C | BAAT | LSM4 | SPARC | CLTC | CAPN8 |
| SOWAHC | POLD4 | GPA33 | TGFB1 | PRC1 | MSLN | CDV3 | RAB26 |
| NCOA7 | CIRBP | SERINC2 | ABHD14A | SNRPD1 | ITGB5 | MED13 | HPN |
| EIF1 | AHCY | CES2 | OCIAD2 | KNSTRN | ALDH1A1 | DST | LMNA |


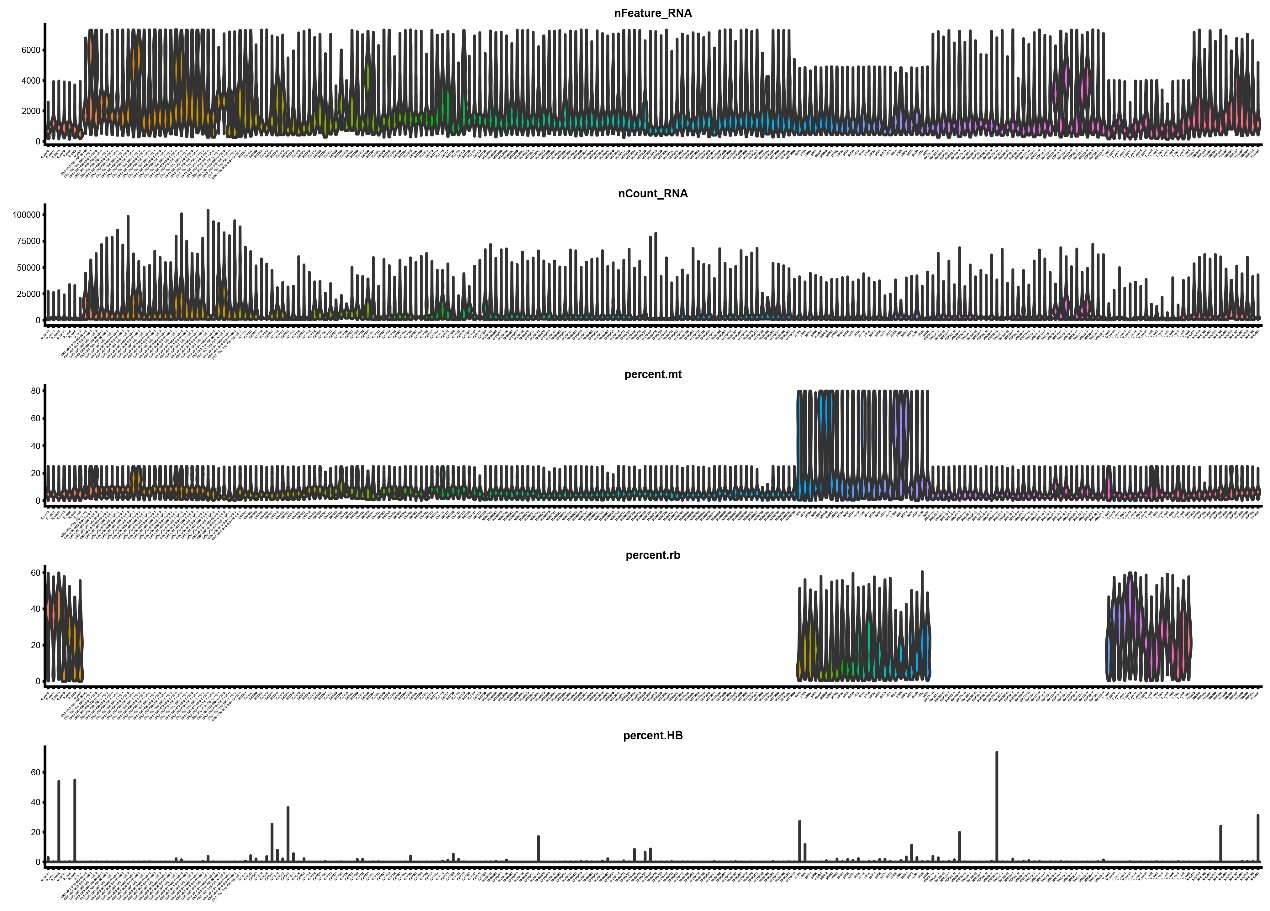


**Fig. S1.** Statistical boxplots of nFeature (Number of Features), nCount (Number of Counts, UMI), percent.mt (Percentage of Mitochondrial Genes), percent.rb (Percentage of Ribosomal Genes), and percent.HB (Percentage of Hemoglobin Genes) for all single-cell sequencing samples before quality control.


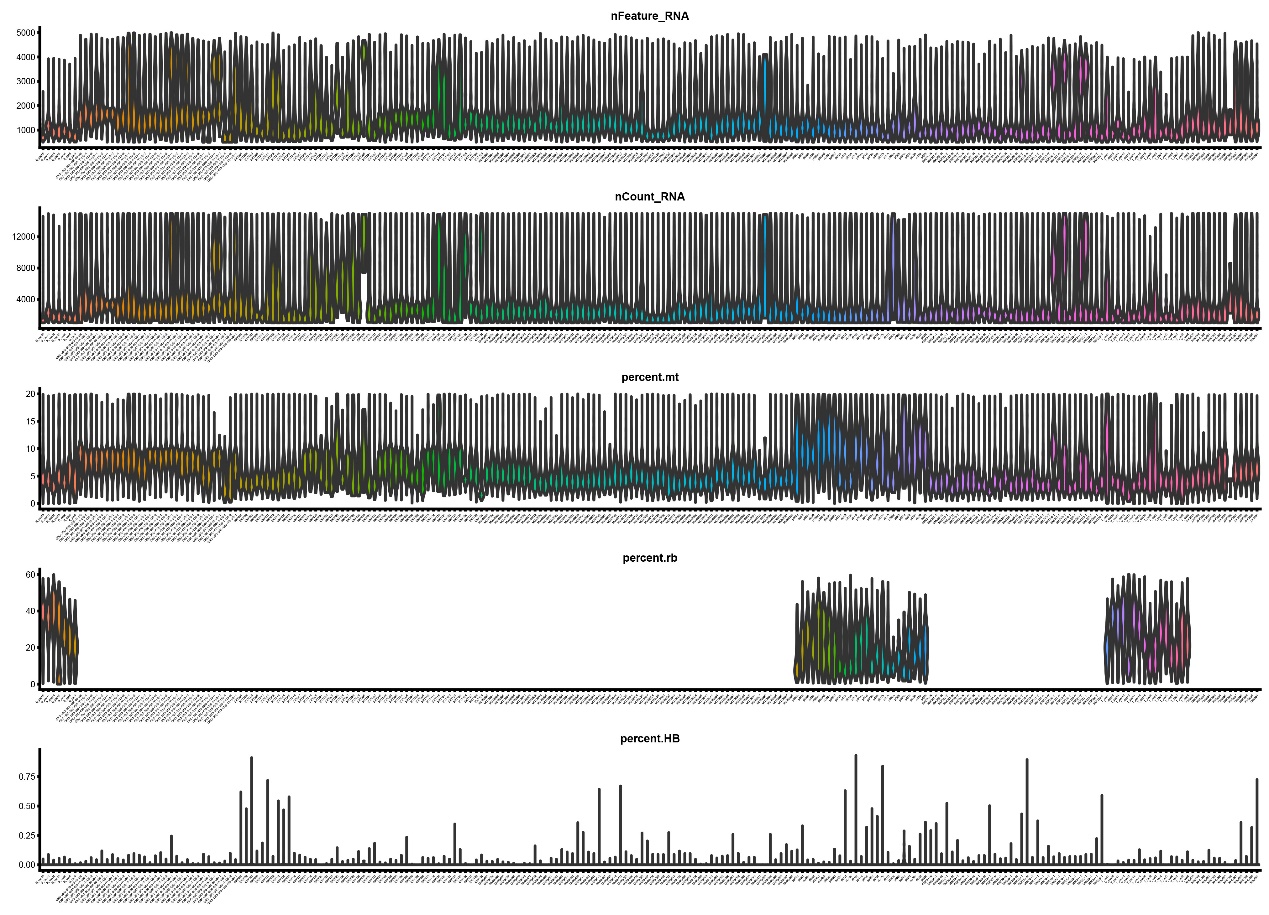


**Fig. S2.** Statistical boxplots of nFeature (Number of Features), nCount (Number of Counts, UMI), percent.mt (Percentage of Mitochondrial Genes), percent.rb (Percentage of Ribosomal Genes), and percent.HB (Percentage of Hemoglobin Genes) for all single-cell sequencing samples after quality control.


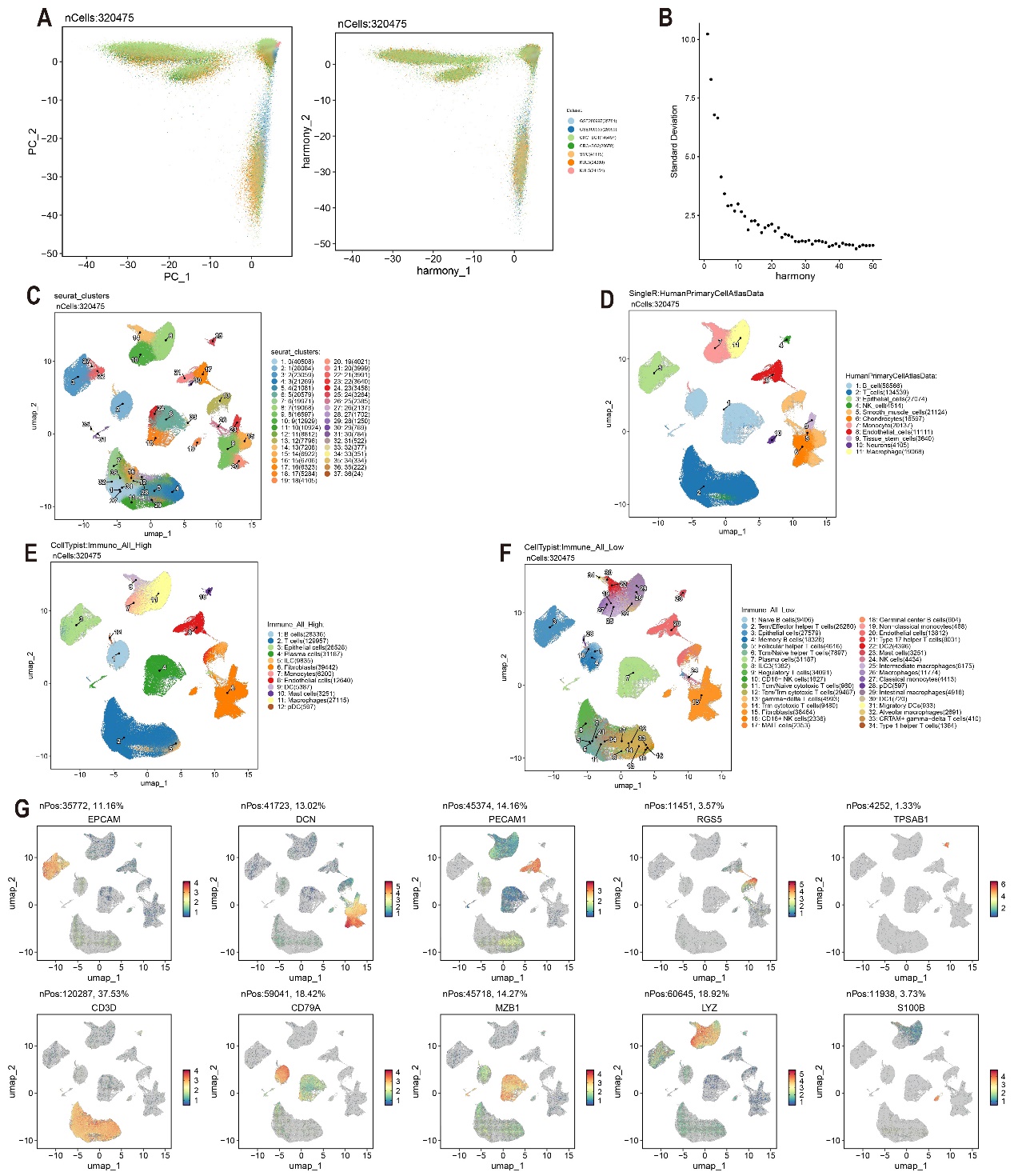


**Fig. S3. First-round dimensionality reduction and annotation results.**A. Cell distribution plot after PCA dimensionality reduction and batch correction using the Harmony algorithm.
B. Anchor plot generated by the Harmony algorithm.
C. UMAP distribution of cell clusters after clustering.
D-F. UMAP cell maps based on SingleR annotation results: annotations using the HumanPrimaryCellAtlasData as the reference dataset, the CellTypist Immune_All_High model, and the CellTypist Immune_All_Low model.
G. UMAP plot of marker gene expression distribution for major cell types.

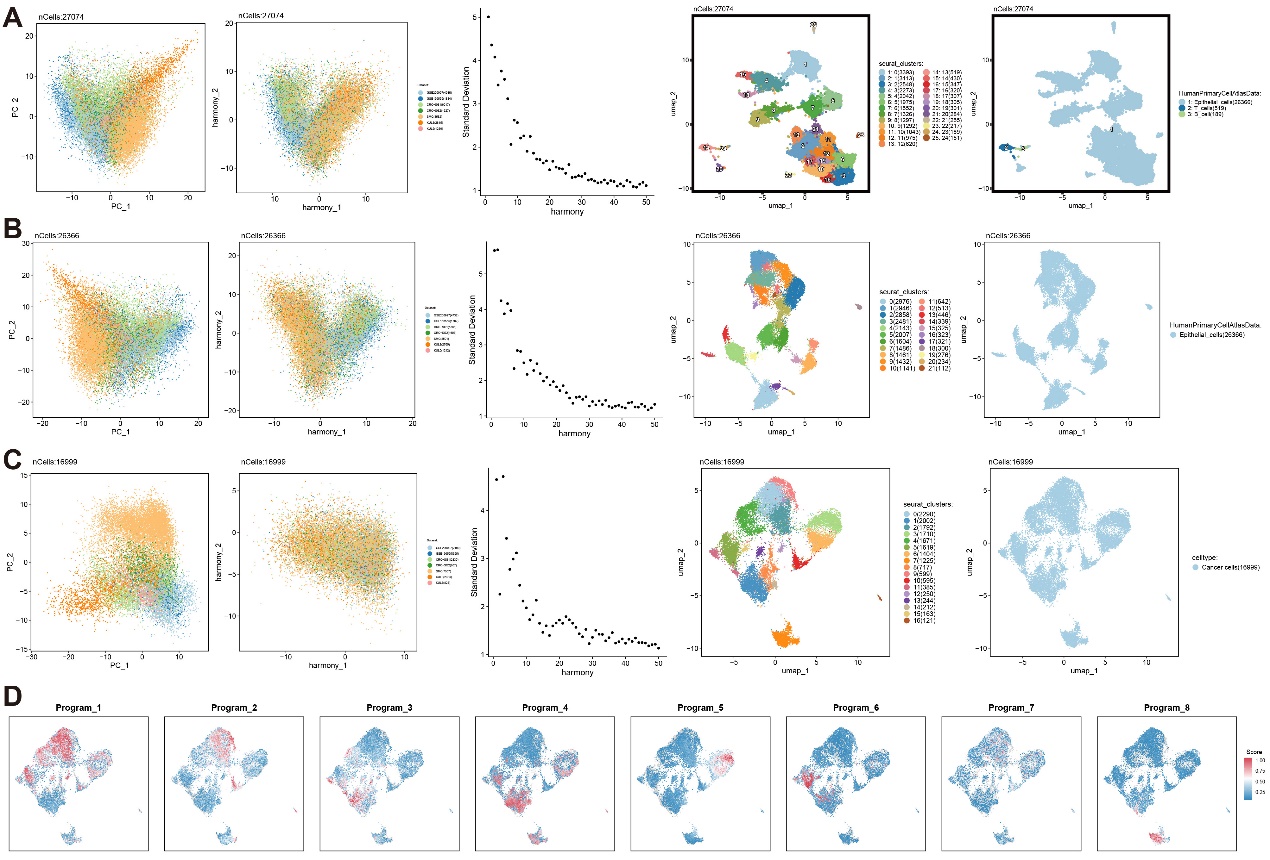


**Fig. S4. Dimensionality reduction and annotation of epithelial cells**A. Epithelial cells extracted from the full cell atlas, with batch effect removal using Harmony, including the Harmony anchor plot, clustering results, and SingleR annotation results.
B. Pure epithelial cells after excluding non-epithelial cells, with batch effect removal using Harmony, including the Harmony anchor plot, clustering results, and SingleR annotation results.
C. Harmony batch effect removal for malignant cells, including the Harmony anchor plot, clustering results, and UMAP distribution map.
D. UMAP plot of the activity distribution of the 8 MCEPs in malignant cells.


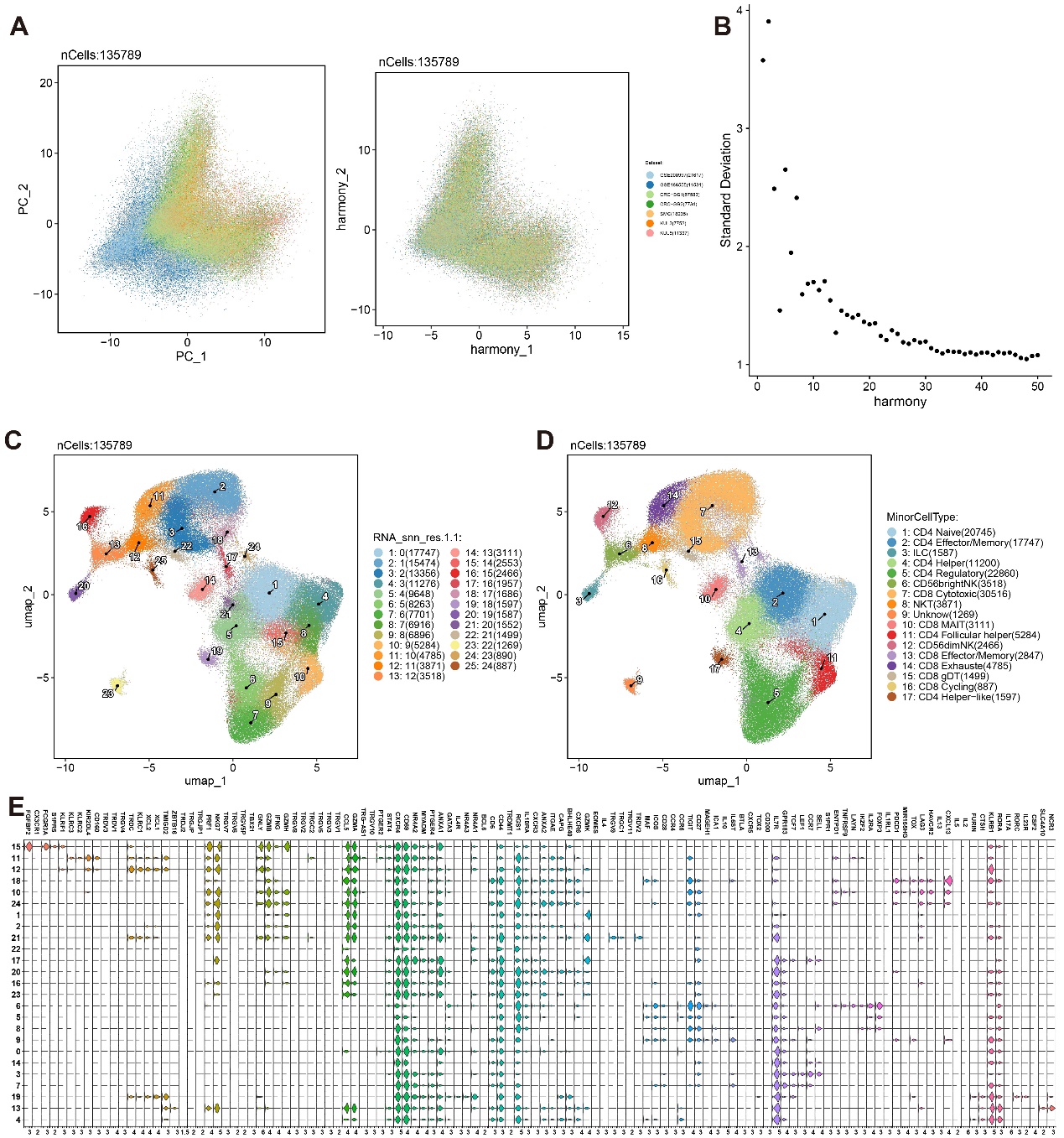


**Fig. S5. Dimensionality reduction and annotation of T/NK cells**A. Cell distribution plot after PCA dimensionality reduction and batch correction using the Harmony algorithm.
B. Anchor plot generated by the Harmony algorithm.
C. UMAP distribution of cell clusters after clustering.
D. Annotation results for T/NK cell subtypes.
E. Expression violin plot of marker genes for each T/NK cell subtype within each cell cluster.


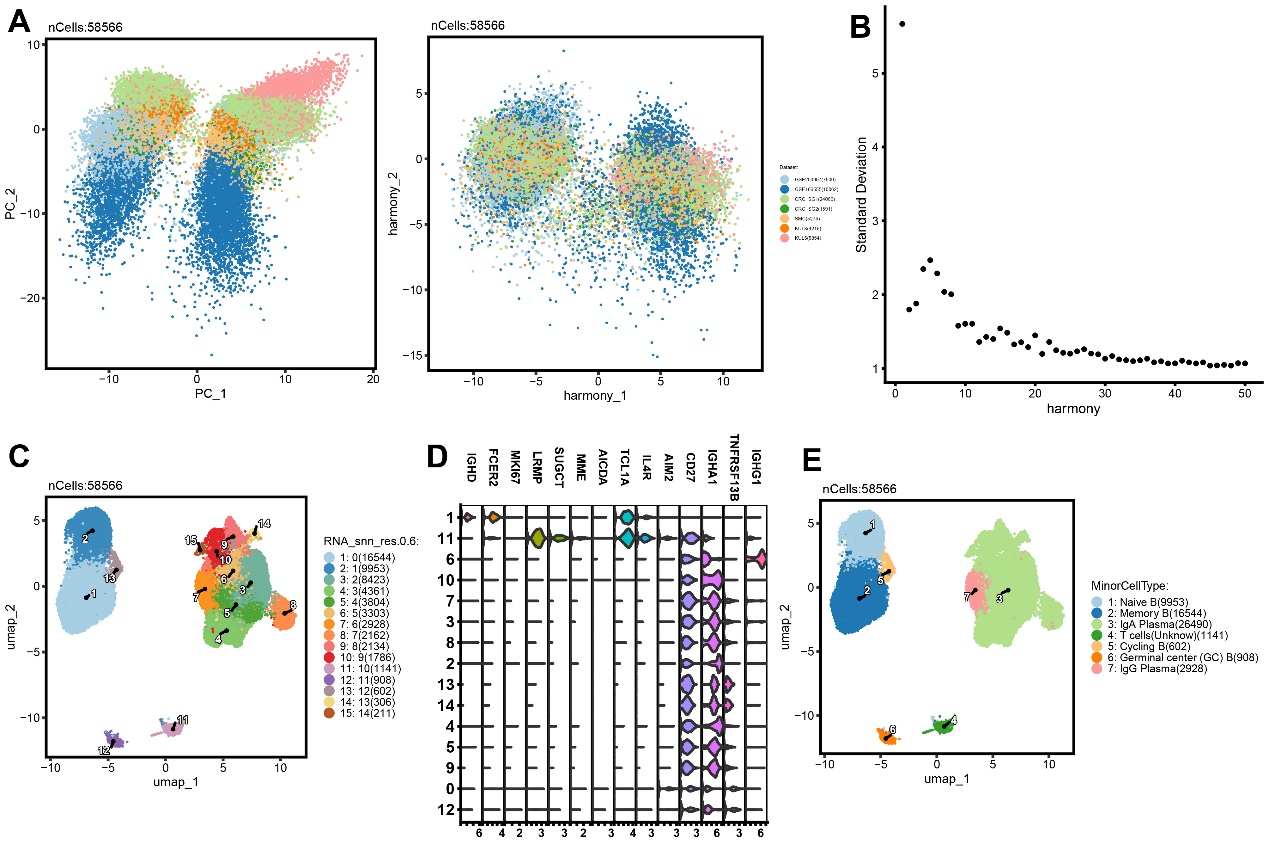


**Fig. S6. Dimensionality reduction and annotation of B/plasma cells**A. Cell distribution plot after PCA dimensionality reduction and batch correction using the Harmony algorithm.
B. Anchor plot generated by the Harmony algorithm.
C. UMAP distribution of cell clusters after clustering.
D. Expression violin plot of marker genes for each B/plasma cell subtype within each cell cluster.
E. Annotation results for B/plasma cell subtypes.


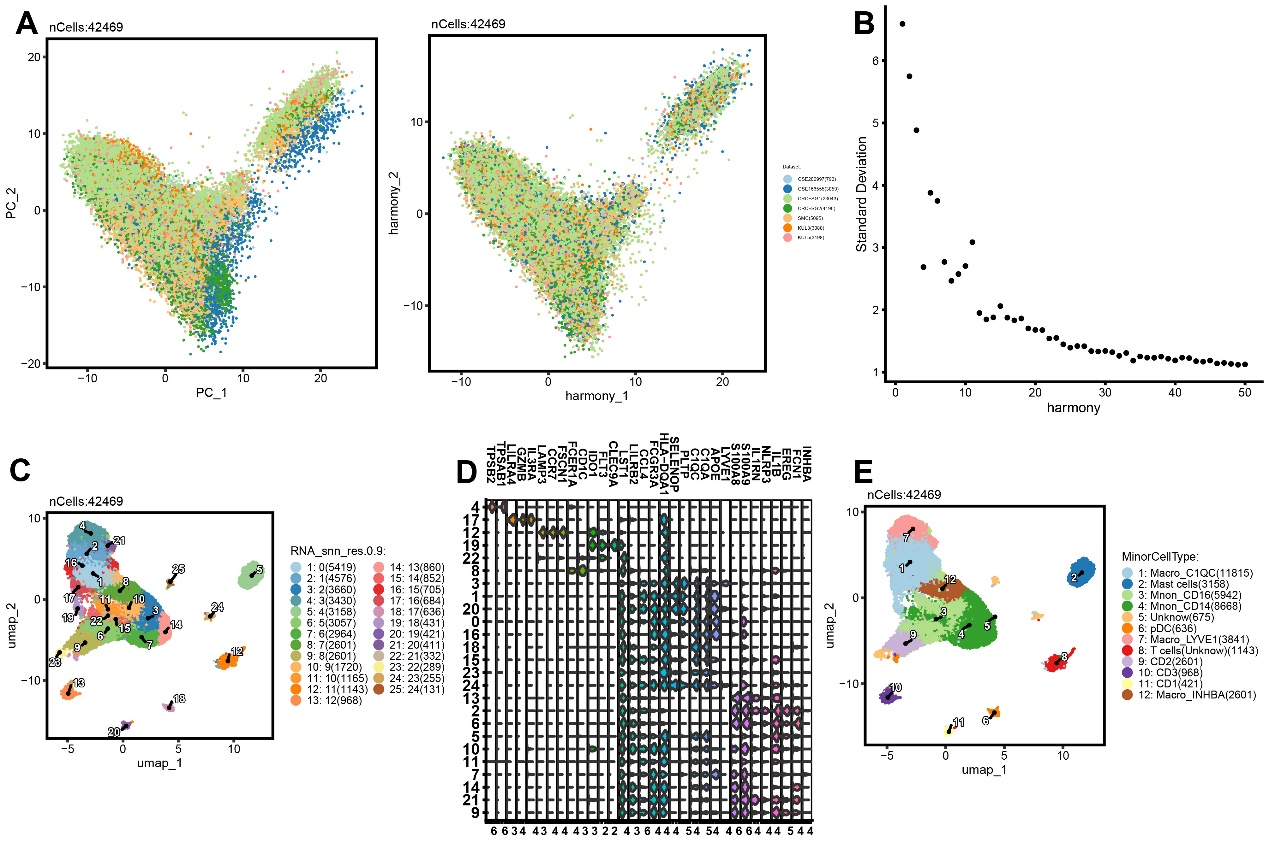


**Fig. S7. Dimensionality reduction and annotation of myeloid cells**A. Cell distribution plot after PCA dimensionality reduction and batch correction using the Harmony algorithm.
B. Anchor plot generated by the Harmony algorithm.
C. UMAP distribution of cell clusters after clustering.
D. Expression violin plot of marker genes for each myeloid cell subtype within each cell cluster.
E. Annotation results for myeloid cell subtypes.


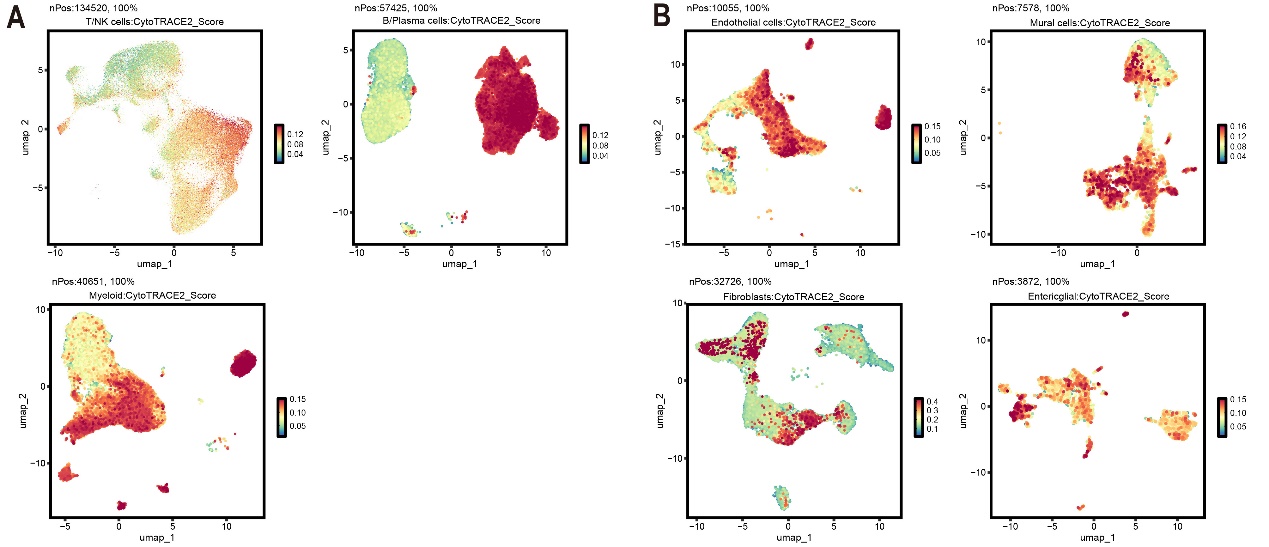


**Fig. S8. CytoTRACE2 stemness scores of immune and stromal cells**A. UMAP map of stemness scores for three types of immune cells: T/NK cells, B/plasma cells, and myeloid cells, where redder colors indicate higher developmental potential.
B. UMAP map of stemness scores for four types of stromal cells: endothelial cells, mural cells, fibroblasts, and enteric glial cells, where redder colors indicate higher developmental potential.


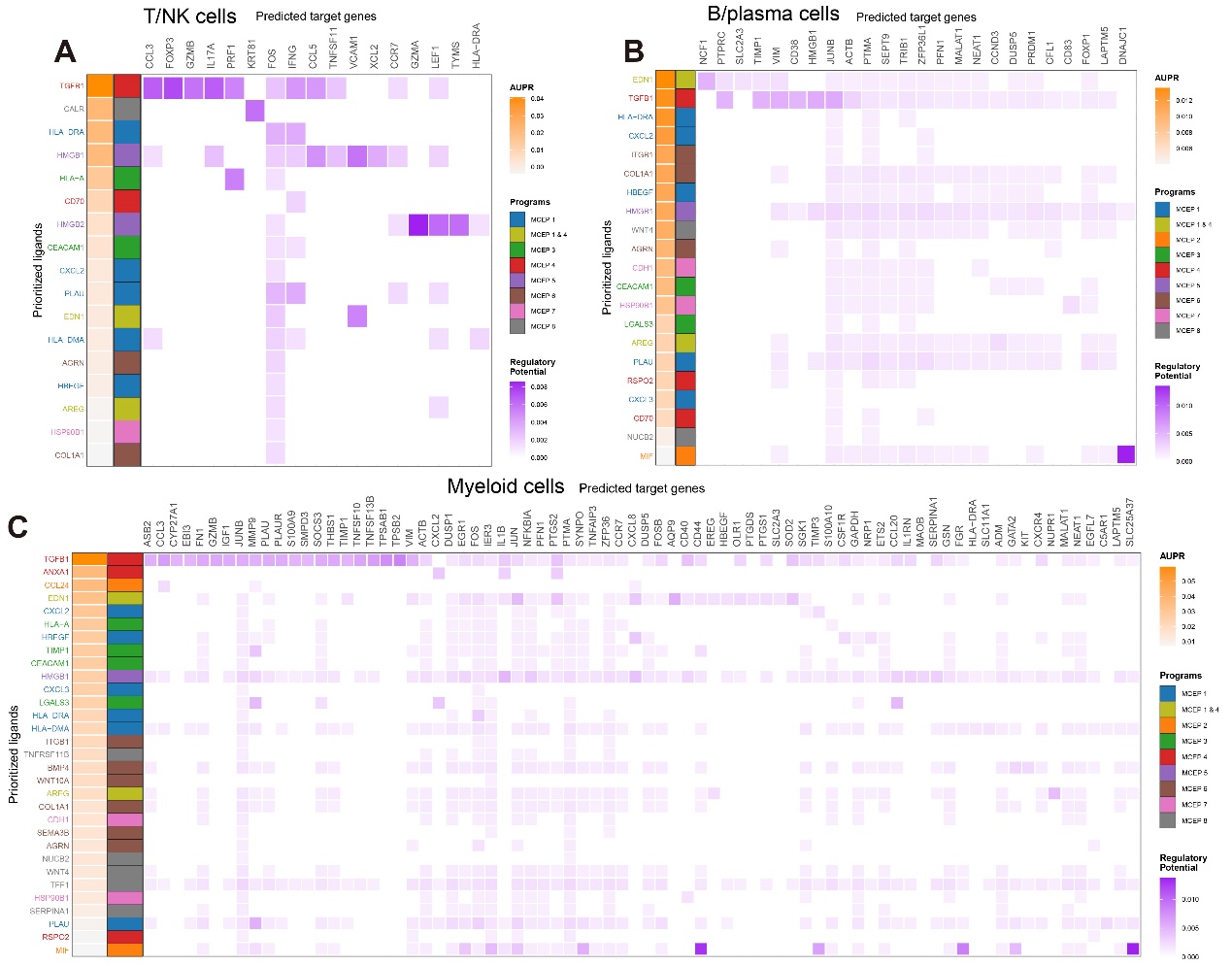


**Fig. S9. Crosstalk of expression programs in immune cells**A. Crosstalk to T/NK cells from different expression programs, with regulators derived from the top 100 weighted genes of the malignant cell expression program, and their corresponding potential target genes in T/NK cells.
B. Crosstalk to B/plasma cells from different expression programs, with regulators derived from the top 100 weighted genes of the malignant cell expression program, and their corresponding potential target genes in B/plasma cells.
C. Crosstalk to myeloid cells from different expression programs, with regulators derived from the top 100 weighted genes of the malignant cell expression program, and their corresponding potential target genes in myeloid cells.


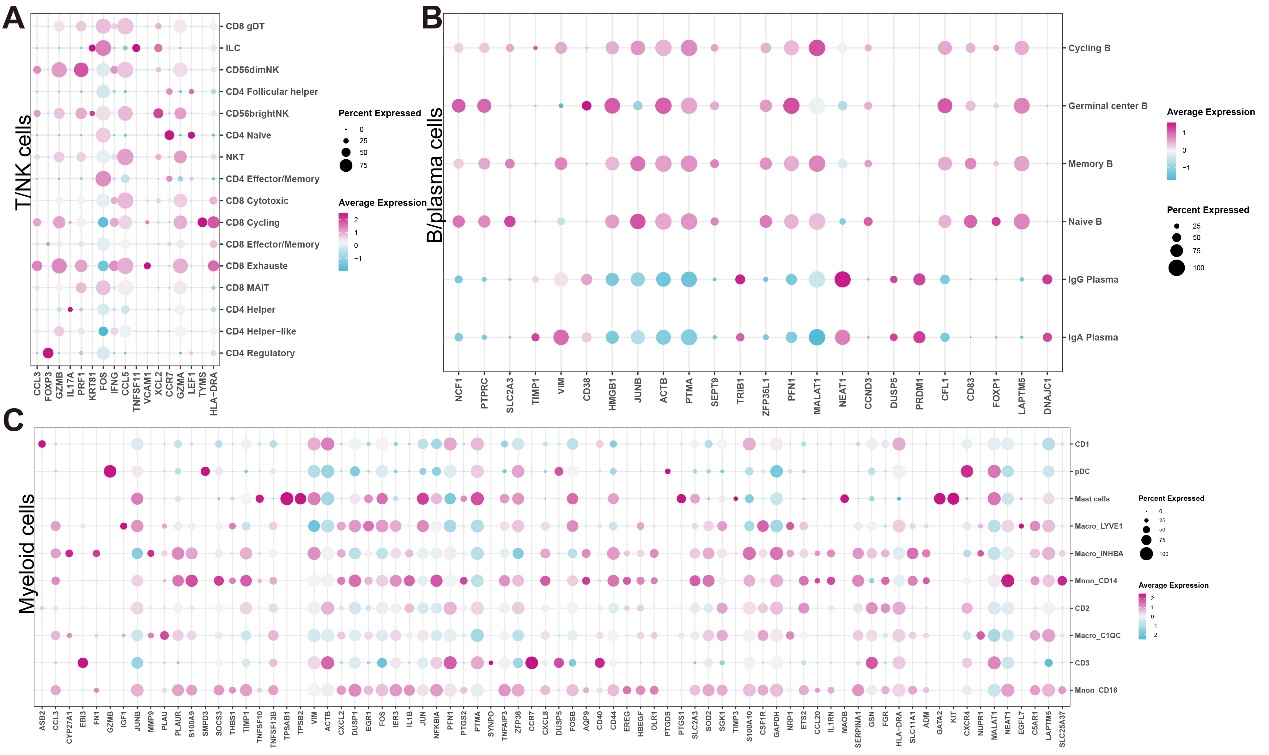


**Fig. S10. Expression of immune cell crosstalk target genes in malignant cell expression programs**A. Expression dot plot of potential regulatory target genes of T/NK cells influenced by the malignant cell expression program across different cell subtypes.
B. Expression dot plot of target genes of B/plasma cells influenced by the malignant cell expression program across different cell subtypes.
C. Expression dot plot of target genes of myeloid cells influenced by the malignant cell expression program across different cell subtypes.


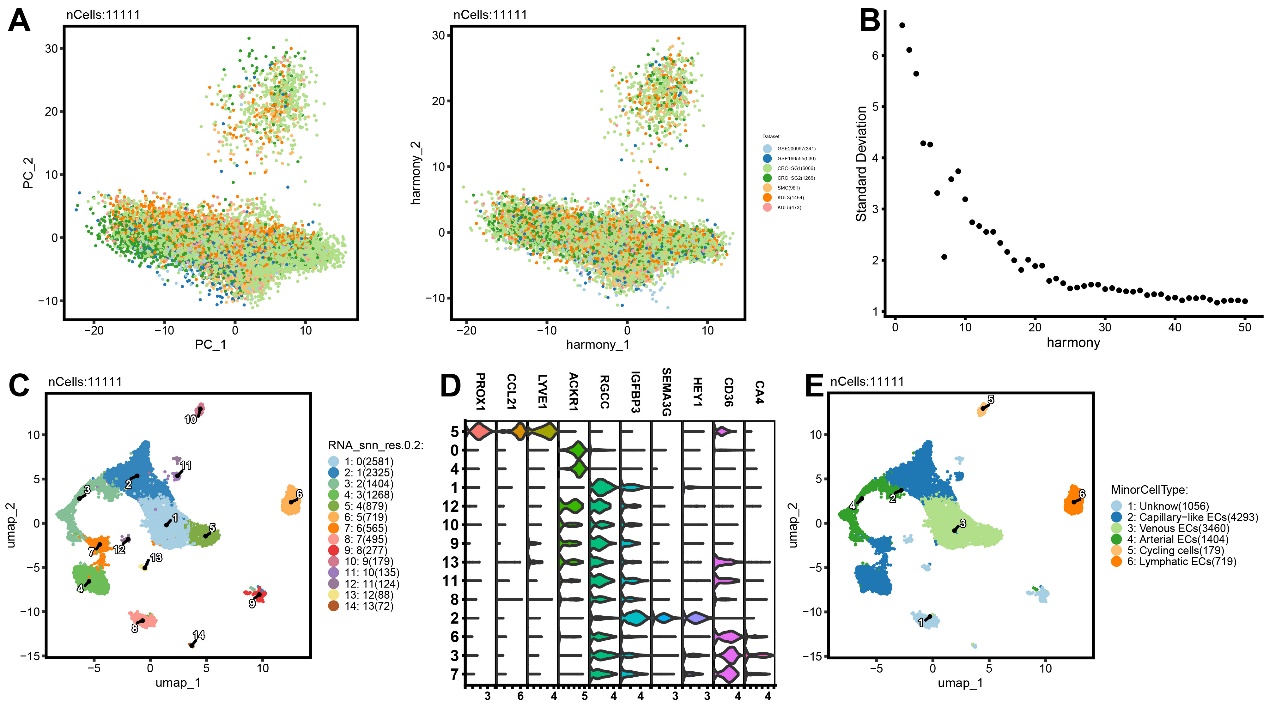


**Fig. S11. Dimensionality reduction and annotation of mural** **cells**A. Cell distribution plot after PCA dimensionality reduction and batch correction using the Harmony algorithm.
B. Anchor plot generated by the Harmony algorithm.
C. UMAP distribution of cell clusters after clustering.
D. Expression violin plot of marker genes for each parietal cell subtype within each cell cluster.
E. Annotation results for parietal cell subtypes.


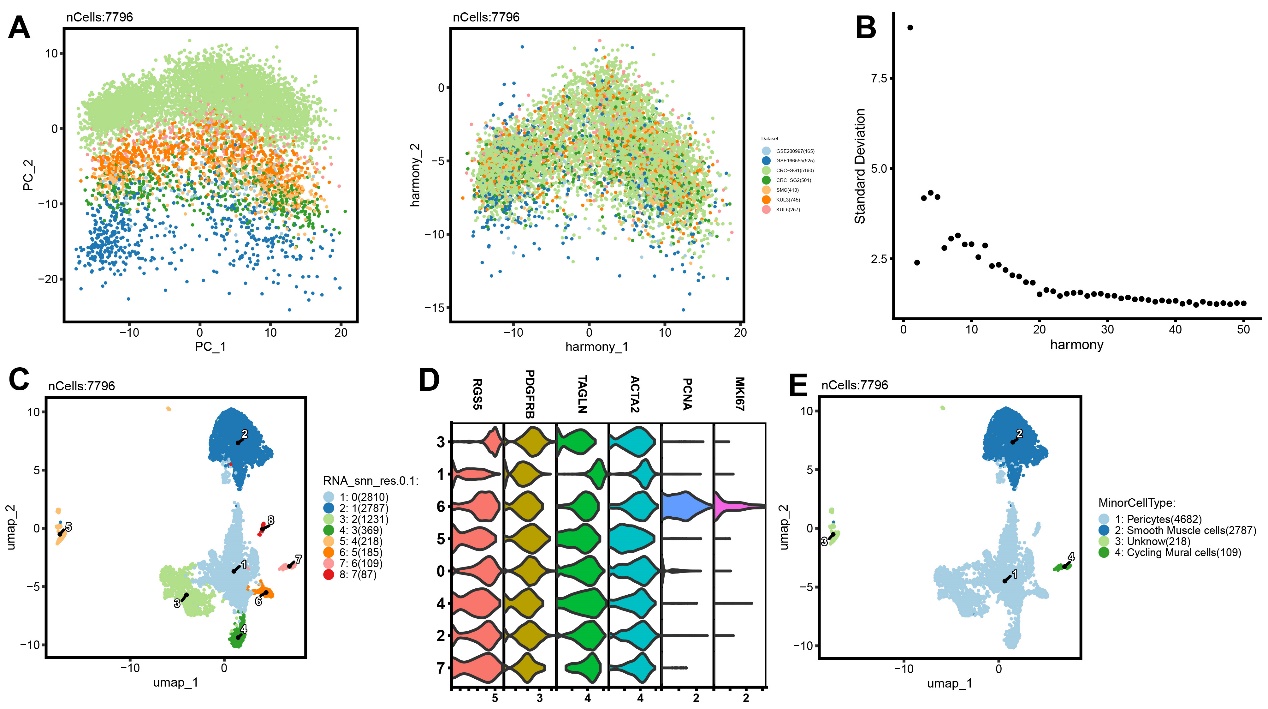


**Fig. S12. Dimensionality reduction and annotation of endothelial cells**A. Cell distribution plot after PCA dimensionality reduction and batch correction using the Harmony algorithm.
B. Anchor plot generated by the Harmony algorithm.
C. UMAP distribution of cell clusters after clustering.
D. Expression violin plot of marker genes for each endothelial cell subtype within each cell cluster.
E. Annotation results for endothelial cell subtypes.


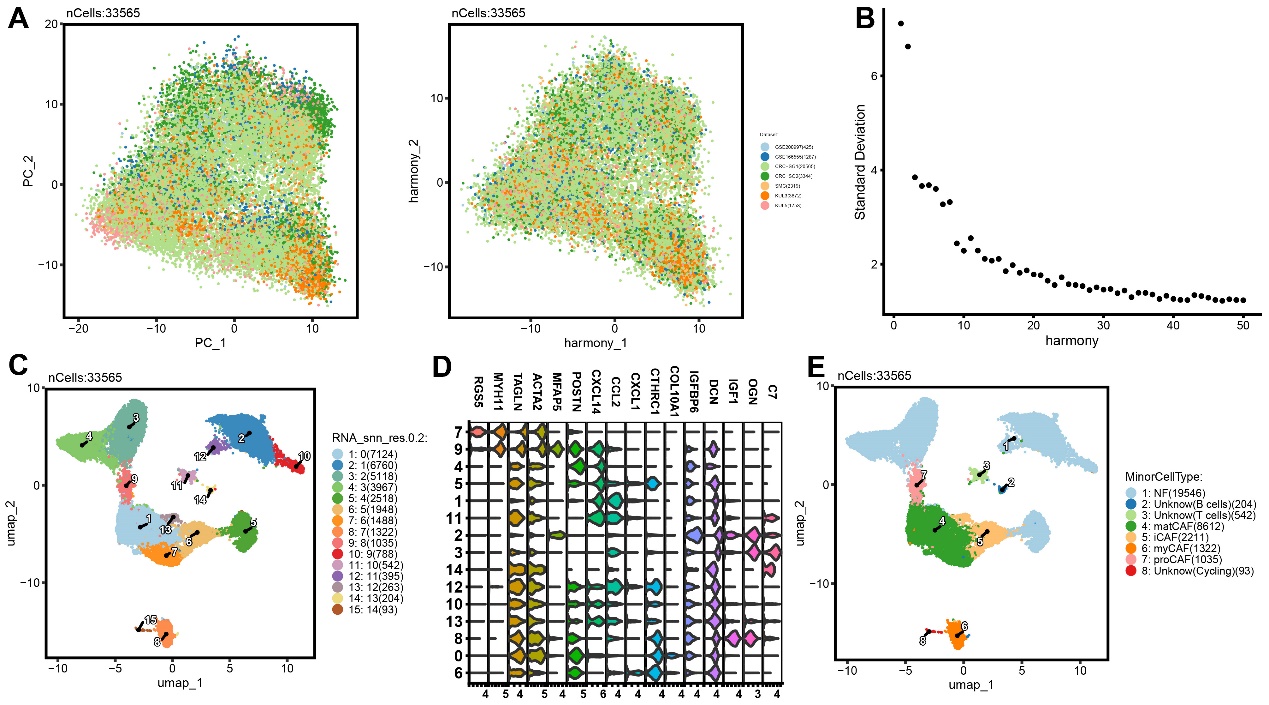


**Fig. S13. Dimensionality reduction and annotation of fibroblasts**A. Cell distribution plot after PCA dimensionality reduction and batch correction using the Harmony algorithm.
B. Anchor plot generated by the Harmony algorithm.
C. UMAP distribution of cell clusters after clustering.
D. Expression violin plot of marker genes for each fibroblast subtype within each cell cluster.
E. Annotation results for fibroblast subtypes.


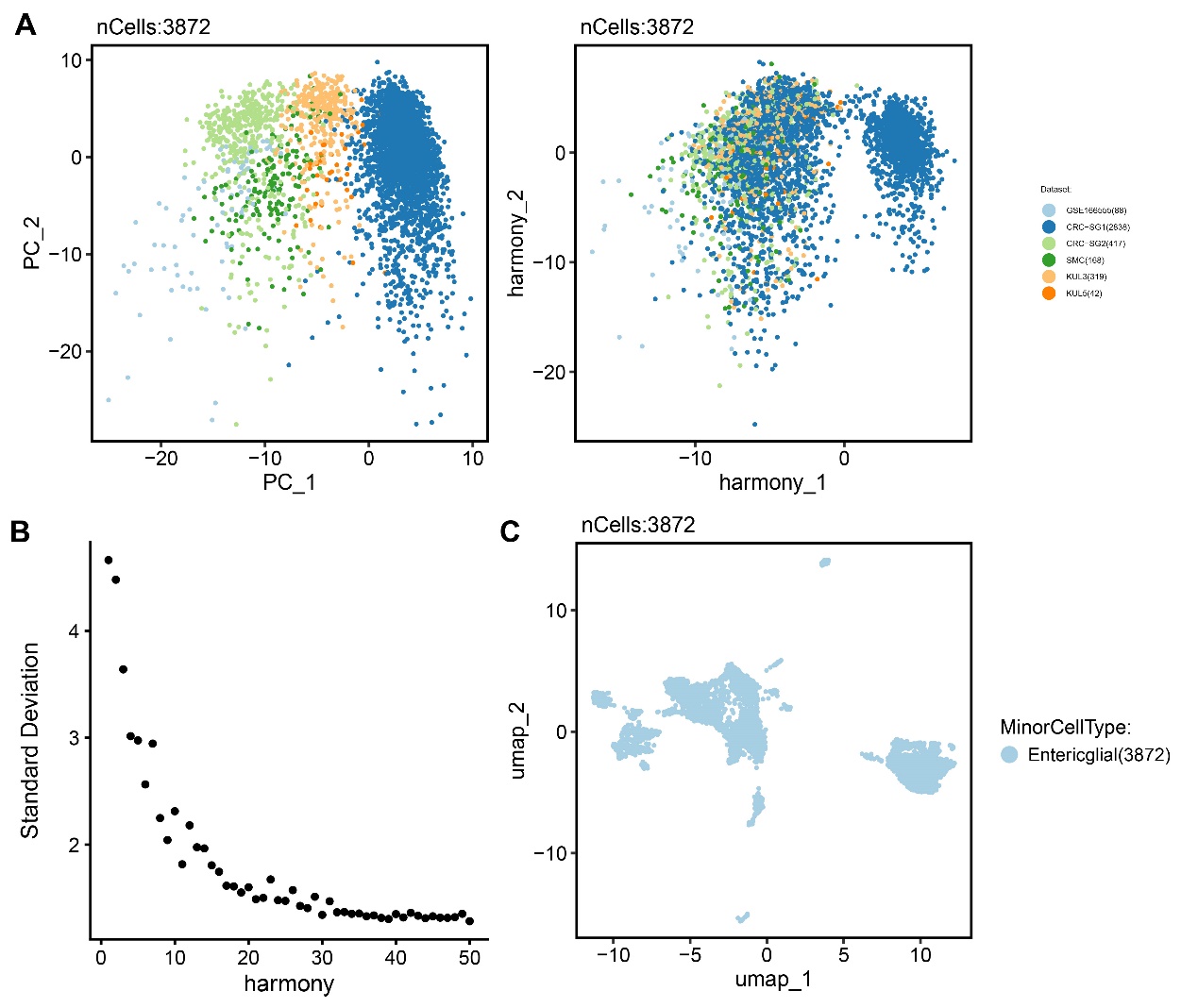


**Fig. S14. Dimensionality reduction and annotation of enteric glial cells**A. Cell distribution plot after PCA dimensionality reduction and batch correction using the Harmony algorithm.
B. Anchor plot generated by the Harmony algorithm.
C. UMAP map of enteric glial cell distribution.


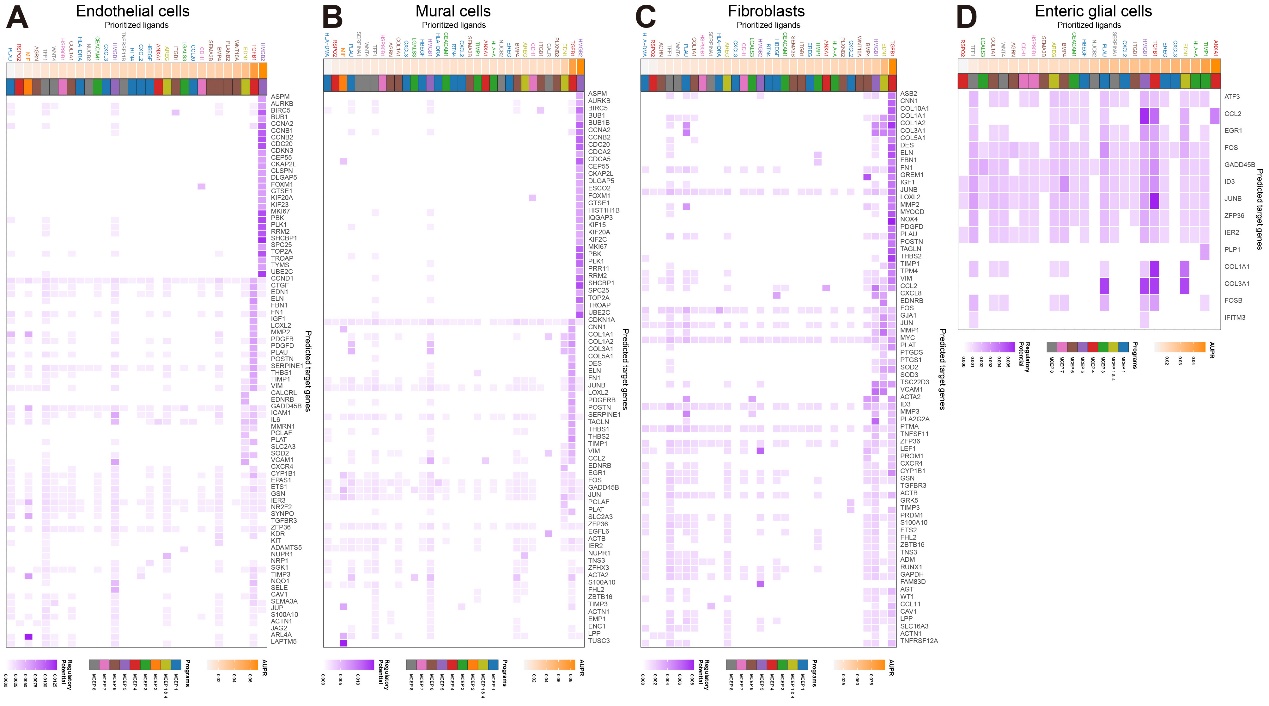


**Fig. S15. Crosstalk of expression programs in stromal cells**A. Crosstalk to endothelial cells from different expression programs, with regulators derived from the top 100 weighted genes of the malignant cell expression program, and their corresponding potential target genes in endothelial cells.
B. Crosstalk to parietal cells from different expression programs, with regulators derived from the top 100 weighted genes of the malignant cell expression program, and their corresponding potential target genes in mural cells.
C. Crosstalk to fibroblasts from different expression programs, with regulators derived from the top 100 weighted genes of the malignant cell expression program, and their corresponding potential target genes in fibroblasts.
D. Crosstalk to enteric glial cells from different expression programs, with regulators derived from the top 100 weighted genes of the malignant cell expression program, and their corresponding potential target genes in enteric glial cells.


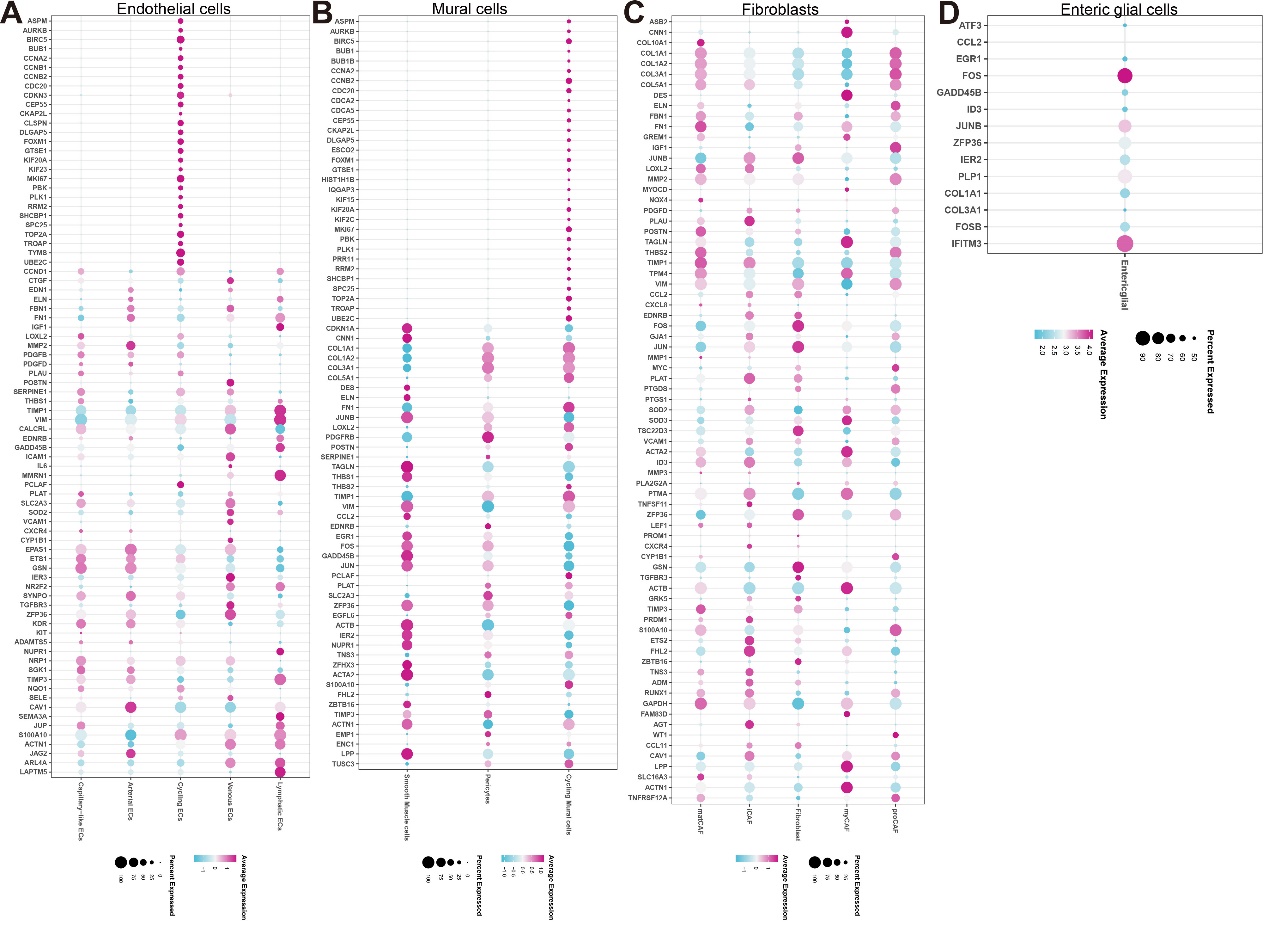


**Fig. S16. Expression of stromal cell crosstalk target genes in malignant cell expression programs**A. Expression dot plot of potential regulatory target genes of endothelial cells influenced by the malignant cell expression program across different cell subtypes.
B. Expression dot plot of target genes of parietal cells influenced by the malignant cell expression program across different cell subtypes.
C. Expression dot plot of target genes of fibroblasts influenced by the malignant cell expression program across different cell subtypes.
D. Expression dot plot of target genes of enteric glial cells influenced by the malignant cell expression program across different cell subtypes.


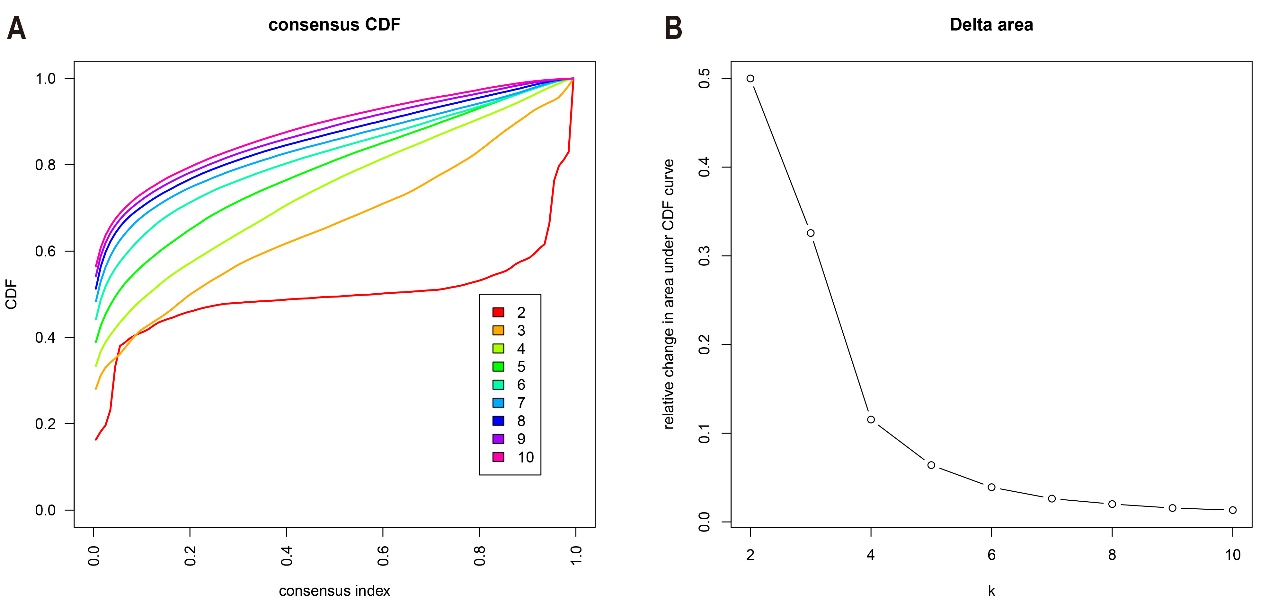


**Fig. S17. Determination of the optimal number of clusters in consensus clustering.**
A. The CDF curves illustrate the consensus distributions for k values ranging from 2 to 10.
B. The area fraction under the CDF curve for k = 2 to k = 10 is shown. The horizontal axis represents the number of clusters (k), while the vertical axis indicates the relative changes in the area under the CDF curves.


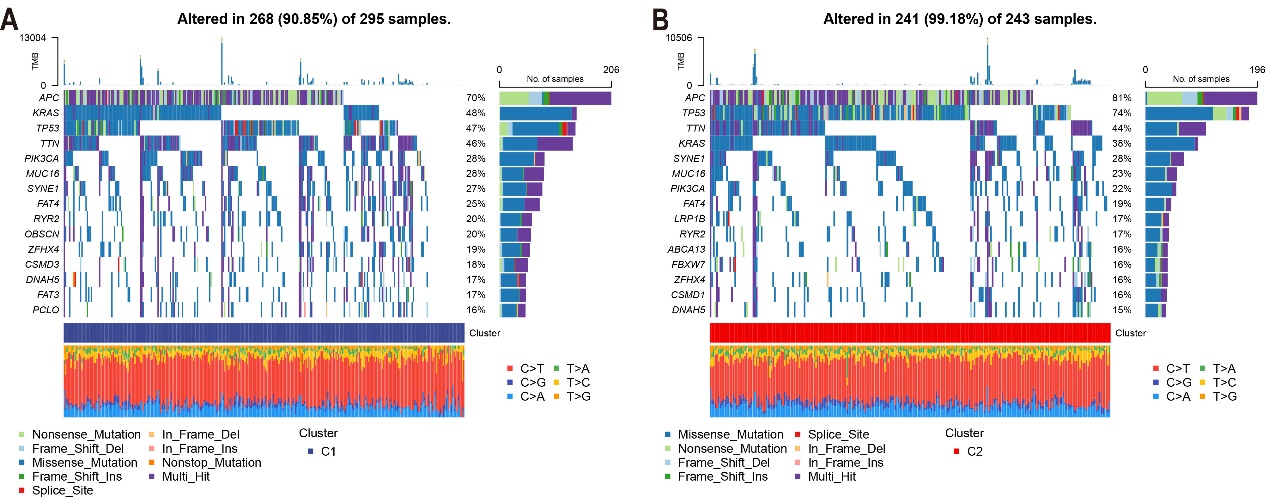


**Fig. S18. Top 15 genes with mutation frequency in each subtype of consensus clustering.**

A-B. Mutation waterfall plots for subtypes C1 and C2, showing frequent mutations.

**Methods and Results**

**1. Major Cell Type Annotation**

We corrected batch effects from different datasets and samples using the Harmony algorithm (Fig. S3A). The results demonstrated effective removal of batch effects. Using the anchor plot from Harmony, we selected the top 30 principal components for UMAP dimensionality reduction and performed clustering analysis, which resulted in the identification of 37 cell clusters (Fig. S3B-C). Subsequently, we performed initial cell type annotation using the HumanPrimaryCellAtlasData reference dataset in SingleR, combined with the Immune_All_High and Immune_All_Low algorithms in CellTypist (Fig. S3D-F). We further validated these cell types through marker genes: EPCAM was highly expressed in epithelial cells, DCN in fibroblasts, PECAM1 in endothelial cells, RGS5 in pericytes, TPSAB1 in mast cells, CD3D in T/NK cells, CD79A in B cells, MZB1 in plasma cells, LYZ in myeloid cells, and S100B in enteric glial cells. These results further supported our annotation of the various cell types^1-4^ (Fig. S3H).

**2. Fine Annotation of T/NK Cells**

We used the Harmony algorithm to correct batch effects in T/NK cells from different datasets and samples (Fig. S5A). Using the anchor plot from Harmony, we selected the top 30 principal components for UMAP dimensionality reduction and performed clustering analysis with a resolution of 1.1, resulting in the identification of 25 cell clusters (Fig. S5B-C). Subsequently, we annotated these clusters into subtypes based on marker genes^5-7^, ultimately identifying 16 distinct T/NK cell subtypes, including CD4 Naïve, CD4 Effector/Memory, and ILC (Fig. S5D).

**3. Fine Annotation of Plasma Cells**

For B cells and plasma cells, we also applied the Harmony algorithm to correct batch effects, and the results demonstrated effective removal of batch effects (Fig. S6A). Using the anchor plot from Harmony, we selected the top 25 principal components for UMAP dimensionality reduction and performed clustering analysis with a resolution of 0.6, resulting in the identification of 15 cell clusters (Fig. S6B-C). Subsequently, we annotated these clusters into subtypes based on marker genes^8^, ultimately identifying 6 distinct subtypes, including Naive B, Memory B, and IgA Plasma cells (Fig. S6E).

**4. Fine Annotation of Myeloid Cells**

In the analysis of myeloid cells, we also used the Harmony algorithm to correct batch effects from different datasets and samples (Fig. S7A). Using the anchor plot from Harmony, we selected the top 30 principal components for UMAP dimensionality reduction and performed clustering analysis with a resolution of 0.9, resulting in the identification of 25 cell clusters (Fig. S7B-C). Subsequently, we annotated these clusters into subtypes based on marker genes^9^, ultimately identifying 11 distinct subtypes, including Macro_C1QC, Mast cells, and Mnon_CD16 (Fig. S7E).

**5. Fine Annotation of Endothelial Cells**

For endothelial cells, we applied the Harmony algorithm for batch effect correction (Fig. S11A). Using the anchor plot from Harmony, we selected the top 25 principal components for UMAP dimensionality reduction and performed clustering analysis with a resolution of 0.2, identifying 24 cell clusters (Fig. S11B-C). These clusters were then annotated into subtypes based on marker genes^10^, resulting in the identification of 5 endothelial cell subtypes, including Capillary-like ECs, Venous ECs, and Arterial ECs (Fig. S11E).

**6. Fine Annotation of Mural cells**

In the analysis of Mural cells, we similarly used the Harmony algorithm for batch effect correction (Fig. S12A). Using the anchor plot from Harmony, we selected the top 25 principal components for UMAP dimensionality reduction and performed clustering analysis with a resolution of 0.1, resulting in the identification of 7 cell clusters (Fig. S12B-C). These clusters were annotated into subtypes based on marker genes^11^, leading to the identification of 3 subtypes: Mural cells, Smooth Muscle cells, and Cycling Mural cells (Fig. S12E).

**7.Fine Annotation of Fibroblasts**

We performed batch effect correction on fibroblasts, with results showing effective removal of batch effects (Fig. S13A). Using the anchor plot from Harmony, we selected the top 30 principal components for UMAP dimensionality reduction and performed clustering analysis with a resolution of 0.2, identifying 15 cell clusters (Fig. S13B-C). These clusters were annotated into subtypes based on marker genes^12^, resulting in the identification of 5 distinct fibroblast subtypes: NF (Normal Fibroblast), matCAF, iCAF, myCAF, and proCAF (Fig. S13E).

**8. Dimensionality Reduction of Enteric Glial Cells**

We performed batch effect correction for enteric glial cells, and the results showed effective removal of batch effects (Fig. S14A). Using the anchor plot from Harmony, we selected the top 20 principal components for UMAP dimensionality reduction (Fig. S14B-C).

**9. Dimensionality Reduction of Endothelial and Malignant Cells**

In the analysis of malignant cells, we first extracted epithelial cells from the complete single-cell atlas. After batch effect correction with Harmony, we used the top 40 principal components to cluster the cells into 25 cell clusters. Subsequent annotation with SingleR identified two non-epithelial subpopulations. After removing these two non-epithelial subpopulations, we performed another round of batch correction and re-clustered the remaining cells using the top 30 principal components into 21 clusters. SingleR annotation revealed that all of these clusters were epithelial cells. Finally, after identifying the malignant cells, batch effect correction was performed again, and the top 40 principal components were used to cluster the malignant cells into 16 distinct cell clusters. All 16 clusters were identified as malignant cells (Fig. S4C).

**References**

^1^ Khaliq, A. M. *et al.*, Refining colorectal cancer classification and clinical stratification through a single-cell atlas. *GENOME BIOL* **23** 113 (2022).

^2^ Qi, J. *et al.*, Single-cell and spatial analysis reveal interaction of FAP(+) fibroblasts and SPP1(+) macrophages in colorectal cancer. *NAT COMMUN* **13** 1742 (2022).

^3^ Wang, F. *et al.*, Single-cell and spatial transcriptome analysis reveals the cellular heterogeneity of liver metastatic colorectal cancer. *SCI ADV* **9** eadf5464 (2023).

^4^ Pfefferle, A. *et al.*, Deciphering Natural Killer Cell Homeostasis. *FRONT IMMUNOL* **11** 812 (2020).

^5^ Zhang, L. *et al.*, Lineage tracking reveals dynamic relationships of T cells in colorectal cancer. *NATURE* **564** 268 (2018).

^6^ Poznanski, S. M. & Ashkar, A. A., Shining light on the significance of NK cell CD56 brightness. *CELL MOL IMMUNOL* **15** 1071 (2018).

^7^ Freud, A. G., Mundy-Bosse, B. L., Yu, J. & Caligiuri, M. A., The Broad Spectrum of Human Natural Killer Cell Diversity. *IMMUNITY* **47** 820 (2017).

^8^ Xia, J. *et al.*, Single-cell landscape and clinical outcomes of infiltrating B cells in colorectal cancer. *IMMUNOLOGY* **168** 135 (2023).

^9^ Cheng, S. *et al.*, A pan-cancer single-cell transcriptional atlas of tumor infiltrating myeloid cells. *CELL* **184** 792 (2021).

^10^ Zheng, Y. *et al.*, Pan-cancer landscape of tumour endothelial cells pinpoints insulin receptor as a novel antiangiogenic target and predicts immunotherapy response. *CLIN TRANSL MED* **13** e1501 (2023).

^11^ Pan, X. *et al.*, Tumour vasculature at single-cell resolution. *NATURE* **632** 429 (2024).

^12^ Chen, B. *et al.*, The molecular classification of cancer-associated fibroblasts on a pan-cancer single-cell transcriptional atlas. *CLIN TRANSL MED* **13** e1516 (2023).
